# The epithelial barrier theory proposes a comprehensive explanation for the origins of allergic and other chronic noncommunicable diseases

**DOI:** 10.1002/1873-3468.70113

**Published:** 2025-07-18

**Authors:** Can Zeyneloglu, Huseyn Babayev, Ismail Ogulur, Sena Ardicli, Yagiz Pat, Duygu Yazici, Bingjie Zhao, Lihong Chang, Xiaoqing Liu, Paolo D'Avino, Manru Li, Ceren Biçer, Fatma Hacer Kurtoğlu Babayev, Raja Dhir, Kari C. Nadeau, Marie‐Charlotte Brüggen, Mubeccel Akdis, Cezmi A. Akdis

**Affiliations:** ^1^ Swiss Institute of Allergy and Asthma Research (SIAF) Davos Switzerland; ^2^ Department of Genetics, Faculty of Veterinary Medicine Bursa Uludag University Turkey; ^3^ Centre for Molecular Medicine, Institute for Vascular Signalling Goethe University Frankfurt am Main Germany; ^4^ Seed Health Inc. Los Angeles CA USA; ^5^ Department of Environmental Health Harvard T.H. Chan School of Public Health Boston MA USA; ^6^ Christine Kühne‐Center for Allergy Research and Education (CK‐CARE) Davos Switzerland; ^7^ Faculty of Medicine University of Zurich Zurich Switzerland; ^8^ Department of Dermatology University Hospital Zurich Zurich Switzerland

**Keywords:** alarmins, epithelial barrier, exposome, noncommunicable diseases, reactive oxygen species, tight junctions

## Abstract

The epithelial barrier theory proposes that modern environmental exposures compromise skin and mucosal surfaces, initiating local inflammation that propagates systemically. The theory integrates epidemiological trends, molecular mechanistic data, and emerging clinical data to show how everyday exposures cause the development and exacerbation of more than 70 chronic noncommunicable diseases. A canonical epithelial cell and barrier injury cascade takes place, generating oxidative stress with increased reactive oxygen species, the release of alarmins, and multiple chemokines and epithelial barrier disruption. The damage to epithelial barriers occurs together with microbial dysbiosis. The alerted immune system fuels various immune activation loops involving multiple cells and chemokines and links barrier leakiness to atopic, autoimmune, metabolic, and neuropsychiatric disease clusters. Supporting the one health concept, abundant exposure of domestic animals and pets to the same groups of toxic substances related to their diseases has recently become more evident. The prevalence of these preventable diseases showed an increase in parallel to industrialization and modernization and epidemiologic exposure to culprit substances throughout the whole world, with a substantial public health burden reaching trillions of dollars each year.

**Impact statement**

Modern environmental exposures compromise epithelial barriers, triggering inflammation, microbial dysbiosis, and chronic disease clusters—atopic, autoimmune, metabolic, neuropsychiatric. These preventable conditions now affect millions and impose a public health and economic burden of trillions annually in healthcare costs and lost productivity.

## Graphical abstract

Exposure to common noxious agents (1), including allergens, pollutants, and micro‐nanoplastics, can cause epithelial barrier damage (2) in our body's protective linings. This may trigger an immune response to our microbiome (3). The epithelial barrier theory explains how this process can lead to chronic noncommunicable diseases (4) affecting organs, such as the gut, lungs, brain, and joints.

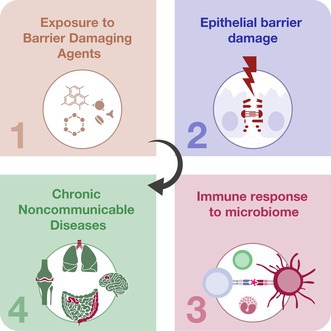

## Abbreviations

**AD**, atopic dermatitis

**AIM2**, absent‐in‐melanoma 2 inflammasome

**AJ**, adherens junction

**AMPK**, AMP‐activated protein kinase

**APAF‐1**, apoptotic protease‐activating factor‐1

**ASC**, apoptosis‐associated speck‐like protein with CARD

**ATM/ATR**, ataxia‐telangiectasia mutated/Rad3‐related kinases

**ATP**, adenosine triphosphate

**BMP**, bone morphogenetic protein

**CaMK II**, calcium/calmodulin‐dependent protein kinase II

**CAR**, coxsackie‐ and adenovirus receptor

**CHOP**, C/EBP homologous protein

**CLE**, confocal laser endomicroscopy

**CXCL/CXCR**, C‐X‐C chemokine ligand/receptor

**DAMP**, damage‐associated molecular pattern

**DNA/dsDNA**, deoxyribonucleic acid/double‐stranded DNA

**DSS**, dextran sodium sulfate (colitis model)

**EIS**, electrical impedance spectroscopy

**EMT**, epithelial‐to‐mesenchymal transition

**ER**, endoplasmic reticulum

**ETC**, electron transport chain

**G‐CSF**, granulocyte colony‐stimulating factor

**GPx4**, glutathione peroxidase 4

**HDAC**, histone deacetylase

**HIF‐1α**, hypoxia‐inducible factor‐1 alpha

**HMGB1**, high‐mobility‐group box 1

**IBD**, inflammatory bowel disease

**IFN**, interferon

**IL**, interleukin

**ILC/ILC2/ILC3**, innate lymphoid cell/group 2/group 3

**JAM**, junctional adhesion molecule

**JNK**, c‐Jun N‐terminal kinase

**LIMK**, LIM‐domain kinase

**MLCK**, myosin light‐chain kinase

**MMP**, matrix metalloproteinase

**MOMP**, mitochondrial outer‐membrane permeabilization

**mROS**, mitochondrial reactive oxygen species

**NCD**, noncommunicable disease

**NF‐κB**, nuclear factor κ‐light‐chain‐enhancer of activated B cells

**NK cell**, natural killer cell

**NLRP3**, NACHT‐LRR‐PYD‐domain protein 3 inflammasome

**OXPHOS**, oxidative phosphorylation

**P2Y13**, purinergic receptor P2Y13

**PEG**, polyethylene glycol

**PM/PM2.5**, particulate matter/≤ 2.5 μm particles

**PPAR**, peroxisome‐proliferator‐activated receptor

**PRR**, pattern‐recognition receptor

**ROS**, reactive oxygen species

**SDS/SLS**, sodium dodecyl sulfate/sodium lauryl sulfate

**SIRTs**, sirtuin deacetylases

**SMA**, smooth‐muscle actin

**SMAC**, second mitochondria‐derived activator of caspases

**TEER**, transepithelial electrical resistance

**TEWL**, transepithelial water loss

**Th1/Th2/Th17**, T‐helper‐1/‐2/‐17 cells

**TJ**, tight junction

**TLR**, Toll‐like receptor

**TNF**, tumor necrosis factor

**TSLP**, thymic stromal lymphopoietin

**UC**, ulcerative colitis

**UPR**, unfolded protein response

**VOCs**, volatile organic compounds

**ZEB**, Zinc‐finger E‐box‐binding protein

**ZO‐1**, zonula occludens‐1

The past six decades have witnessed a dramatic and concerning rise in the prevalence of many chronic noncommunicable diseases (NCDs), reaching pandemic proportions globally [1]. Seeking explanations for this trend, research beginning around 2000, particularly focusing on the mechanisms of type 2 inflammatory diseases like eczema, asthma, and chronic rhinosinusitis (CRS), highlighted the critical role of epithelial cell death and subsequent chronic barrier defects. These initial studies revealed that epithelial dysfunction led to peri‐epithelial inflammation driven by both innate and adaptive immunity [2, 3, 4].

Early understanding framed epithelial barrier functions primarily as ‘keeping away’ harmful agents like allergens, toxins, pollutants, and microbes by acting as a physical, chemical, and immunological barrier. Additional concepts included ‘washing away’ the inflammation by opening epithelial barriers and draining the inflammatory cells and cytokines to the lumens of mucosal surfaces and outside the skin, and ‘suppression’ of inflammation via regulatory cells and cytokines active during type 2 inflammation or high‐dose allergen exposure in healthy individuals [5, 6]. The following studies demonstrated compromised epithelial barriers in conditions like asthma, atopic dermatitis (AD), and CRS, exposure to barrier‐toxic substances, and the effects of type 2 immune responses, particularly cytokines IL‐4 and IL‐13 [7, 8, 9, 10]. Genetic predispositions have been suggested in all of these diseases, which may form the background for increased inflammation or barrier defects; however, they alone are not sufficient to explain the substantial increase after the 1960s and 2000s [11].

**Fig. 1:**
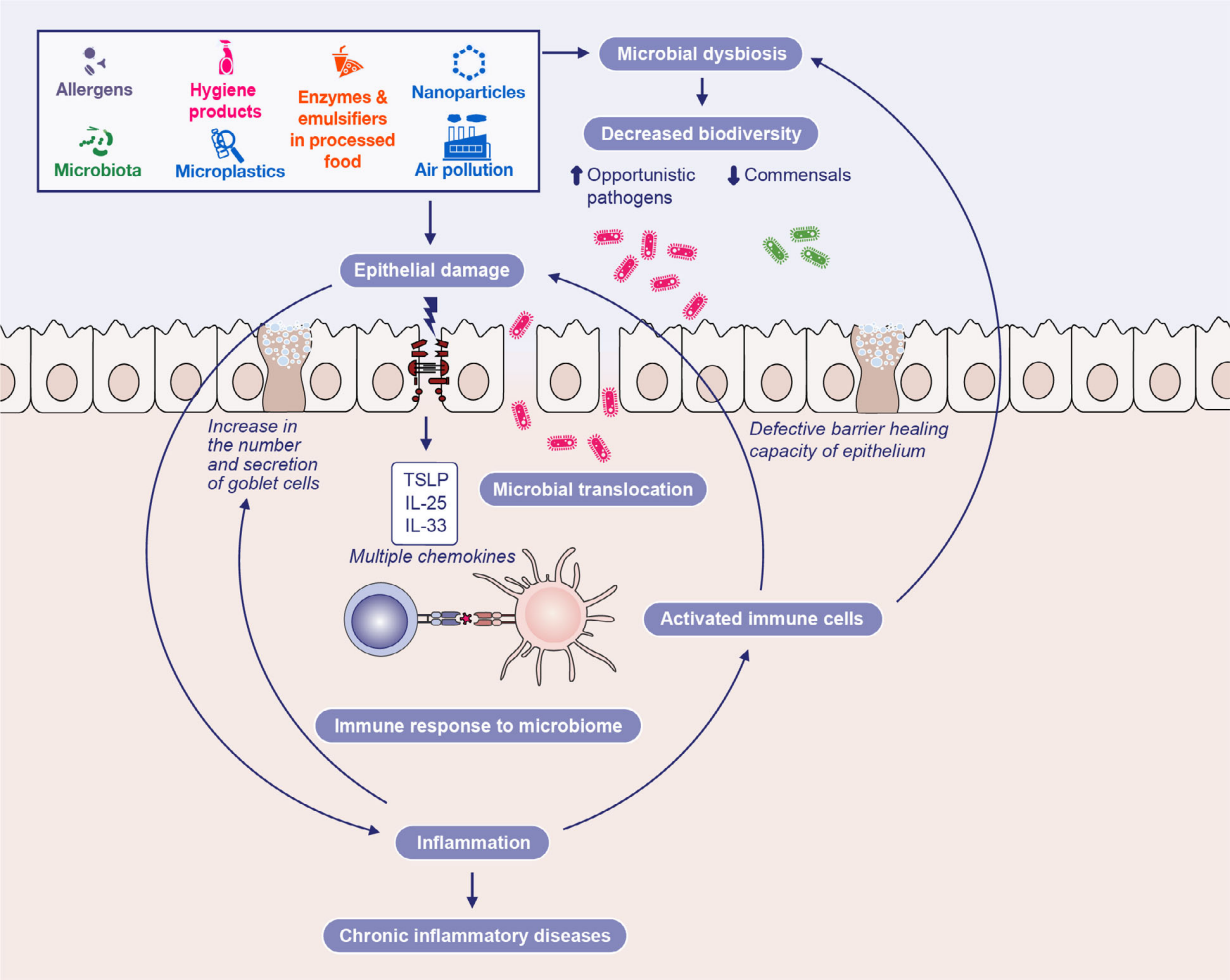
Epithelial barrier damage caused by multiple damaging substances leads to the release of alarmins, microbial dysbiosis and subepithelial infiltration of commensal and dysbiotic microorganisms. The resulting immune response triggers inflammation, which leads to further epithelial barrier damage and the propagation of a vicious cycle. The loss of tolerance to microorganisms, activation of effector immune cells and release of pro‐inflammatory cytokines into the circulation may decrease the threshold/trigger multiple chronic inflammatory diseases at distant sites.

Building on this foundation, subsequent work identified specific lifestyle‐related and environmental substances capable of damaging epithelial barriers, including common detergents, household cleaning substances, chlorine, toothpaste, food emulsifiers, pesticides, air pollutants and micro‐nanoplastics. These agents were shown to induce epithelial damage, trigger the release of alarmins, and provoke tissue inflammation [12, 13, 14, 15, 16, 17, 18, 19, 20, 21, 22]. Compromise of these critical barriers not only triggers local pathological processes, but can also lead to systemic consequences as damaging agents, microbial components, or inflammatory mediators disseminate from the initial site of injury. This systemic spread is crucial for understanding the link between localized epithelial damage and the development or exacerbation of diseases in distant organs (Table 1). This body of work culminated in the formulation of the broader ‘epithelial barrier theory’. This theory posits that widespread exposure to environmental agents linked to industrialization, urbanization, and modern life compromises the integrity of epithelial barriers [1]. This dysfunction often occurs alongside microbial dysbiosis—characterized by a decrease in beneficial commensal microbes and colonization by opportunistic pathogens—and facilitates bacterial translocation into deeper tissues, initiating the cascade of dysbiosis, immune responses, and inflammation central to the epithelial barrier theory (Fig. 1). The resulting tissue and systemic inflammation, coupled with immune dysregulation, are proposed as key underlying factors contributing to the increasing prevalence and exacerbation of allergic, autoimmune, metabolic, neuroimmune, and other chronic inflammatory diseases [1, 23]. Despite growing awareness, there remains a significant need to advance the scientific understanding of the factors and molecular pathways involved in epithelial barrier leakiness and to inform policymakers about the potential detrimental effects of implicated substances.

**Table 1:** Systemic translocation and distant organ effects of selected effectors following epithelial barrier damage.

Effectors	Primary barrier site(s) of entry/damage	Evidence/mechanism of systemic translocation or spread	Examples of distant organs/systems affected & observed/hypothesized effects	Selected key references
Micro‐ and nanoplastics (e.g., polystyrene particles)	Gastrointestinal tract, airways, skin (potential)	Direct epithelial penetration (e.g., M‐cell uptake in the gut); potential lymphatic and bloodstream dissemination following breach; documented transplacental transfer	Placenta: Accumulation within placental tissue Liver: Hepatotoxicity, inflammation, metabolic dysregulation (potentially via gut–liver axis) Other organs (potential): Spleen, kidney, brain (based on particle biodistribution); induction of systemic inflammation, oxidative stress in various tissues	[19, 58, 95, 111, 136]
Particulate matter (PM) (from air pollutants)	Lungs, airways, nasal epithelium	Translocation of ultrafine particles from alveoli into systemic circulation; potential olfactory nerve transport from the nasal cavity to the brain	Cardiovascular system: endothelial dysfunction, atherosclerosis, hypertension Brain: Neuroinflammation, potential contribution to neurodegenerative diseases (e.g., multiple sclerosis), cognitive impacts Liver: Inflammation, fibrosis (potentially involving gut–lung–liver axis interactions) Systemic: Increased chronic systemic inflammation and oxidative stress	[25, 134, 152]
Microbial products & translocated microbes (e.g., LPS, PAMPs, whole bacteria/fungi from dysbiotic microbiota)	Gastrointestinal tract (‘leaky gut’), airways, skin	Compromised epithelial tight junctions and overall barrier integrity leading to passage of microbial components or whole microbes into the submucosa, lymphatics, and systemic circulation	Liver: Nonalcoholic fatty liver disease (NAFLD/NASH), autoimmune hepatitis, fibrosis (via gut–liver axis) Joints: Inflammatory arthritis (e.g., rheumatoid arthritis, ankylosing spondylitis via gut–joint axis) Brain: Neuroinflammation, anxiety, depression, autism spectrum disorders, Parkinson's disease, Alzheimer's disease (via gut–brain axis) Pancreas/islets: Autoimmune diabetes (Type 1) Adipose Tissue: Inflammation, formation of ‘creeping fat’ in IBD Systemic: Chronic low‐grade inflammation, metabolic syndrome, development/exacerbation of autoimmune diseases	[1, 24, 56, 183, 216, 217, 218, 219, 220, 221, 222, 223, 224]
Alarmins (IL‐33, TSLP, IL‐25, other DAMPs), Pro‐inflammatory Cytokines (IL‐1β, TNF‐α, IL‐6)	Any damaged epithelial site (skin, gut, airways)	Release from damaged/activated epithelial cells and resident immune cells into local tissue microenvironment, with subsequent spillover into systemic circulation	Systemic: Contribution to and maintenance of chronic low‐grade systemic inflammation; priming or exacerbation of inflammatory responses in distant organs/tissues (e.g., demonstrated gut–lung axis and skin–gut axis effects in allergic inflammation); modulation of systemic immune cell activity and trafficking; fever; acute phase protein responses	[1, 16, 18, 20, 21, 57, 86, 139, 182, 190, 234, 235, 246]

Recent research has identified 68 diseases that align with this theory [23]. Many of these conditions, such as asthma, AD, CRS, allergic rhinitis, eosinophilic esophagitis (EoE), inflammatory bowel disease (IBD), and celiac disease, are characterized by inflammation localized to the affected organ's epithelial tissue. Another category includes metabolic and autoimmune diseases like obesity, diabetes mellitus, rheumatoid arthritis, multiple sclerosis, fatty liver disease, autoimmune hepatitis, systemic lupus erythematosus, and ankylosing spondylitis. While these conditions manifest systemically or in organs distant from primary barrier sites, they are often associated with chronic mucosal inflammation involving gut or lung barrier defects or conditions like periodontitis [1, 24, 25]. These are thought to be mediated through inter‐organ axes like the gut–thyroid, gut–joint, gut–liver, and gut–brain pathways, contributing to diseases, such as Hashimoto's thyroiditis, Graves' disease, osteoarthritis, obesity, diabetes, and liver cirrhosis [23]. Furthermore, accumulating evidence suggests a link between increased intestinal barrier permeability (‘leaky gut’) and neuropsychiatric disorders, including Parkinson's disease, Alzheimer's disease, stress‐related psychiatric conditions, autism spectrum disorders, and chronic depression [1].

Athletes and highly active individuals are exposed to numerous factors that can compromise epithelial barrier integrity and microbiome health. Intense physical exertion stresses multiple organs, leading to inflammation and tissue damage [26]. Harmful exposures include cleaning agents, chlorine in pools, air pollutants, microplastics, and additives in processed high‐calorie sports foods. These factors, combined with sleep disturbances, frequent travel, and hygienic stressors, heighten the risk of barrier dysfunction, particularly in the skin, gut, and respiratory tract [27]. Athletes in winter and water sports are especially vulnerable due to high exposure to airborne and chlorinated substances [27].

While moderate exercise improves microbiota diversity, enhances gut barrier function, and reduces inflammation that benefits conditions like cardiovascular disease, diabetes, and obesity [28, 29, 30, 31, 32, 33, 34, 35], elite‐level physical stress may reverse these advantages [36, 37]. Exercise‐induced oxidative stress, immune shifts (e.g., increased Th2 and reduced Th1/Treg), and gastrointestinal symptoms reflect the vulnerability of overstressed athletes [38]. Despite these risks, elite athletes who maintain optimal hygiene, nutrition, and recovery can mitigate some damage [39, 40].

The well‐being of humans, animals, and the environment is inherently interconnected, as acknowledged by the ‘One Health Concept’, an integrated and holistic approach designed to sustainably balance and optimize the health of people, animals, and ecosystems [41, 42, 43]. The conceptual framework of the epithelial barrier theory can be effectively extended to domestic animals within an integrative scope [41]. This issue is especially critical in livestock species, considering the high consumption of animal‐derived products as a major protein source in human nutrition. Furthermore, companion animals living in close proximity to humans are similarly (and in some cases more extensively) exposed to chemicals and environmental pollutants associated with modernization and urbanization, such as detergents, surfactants, air pollutants, and food additives, including a high and increasing burden of micro‐nanoplastics exposure [41].

Notably, large‐scale analyses have identified skin disorders and enteropathies as common conditions in dogs [44], highlighting the potential contribution of chemical‐induced epithelial barrier disruption to their rising prevalence. Due to their barefoot contact with household surfaces, especially via furless paw pads, they may absorb higher doses of these substances [45]. Conditions like canine AD, often affecting high‐contact areas like paws and axillae, support this risk [41]. Pets also face chemical exposure through grooming behaviors, such as licking paws or using pet toothpaste containing surfactants like sodium lauryl sulfate (SLS). Additionally, animals are vulnerable to airborne pollutants, including ozone, volatile organic compounds, and microplastics, particularly those closer to ground level, such as small dog breeds [41]. Indoor environments pose continuous exposure risks, especially for exclusively indoor animals like cats. The widespread use of plastic‐based pet products and frequent cleaning in multi‐pet households further heighten exposure. Moreover, poisoning from insecticides, rodenticides, and household chemicals remains a consistent threat, with several reports documenting such toxic events in domestic animals [46, 47]. Addressing environmental pollution and related risks to human and animal health requires a coordinated, collaborative, cross‐sectoral action and a global strategy within the One Health Framework [48].

This article synthesizes current evidence on the cellular and molecular mechanisms by which environmental stressors compromise epithelial barriers. It elucidates how modern toxins disrupt barrier integrity and traces these disturbances to the development of more than 70 chronic NCDs and their immunological sequelae. The discussion is framed within the ‘One Health’ paradigm, underscoring the cross‐species importance of safeguarding epithelial barrier function for human and animal health alike.

## Substances that cause epithelial barrier damage

After the chemistry revolution, urbanization and modernization have introduced widespread human exposure to numerous chemicals and substances since the 1960s (Table 2). A significant development was the introduction of potent anionic surfactants and enzymes into laundry detergents to enhance cleaning, which coincided with epidemiological observations linking occupational detergent exposure to asthma, rhinitis, and AD [1]. Experimental studies investigating the effects of highly diluted laundry detergent, even 50 000‐times dilution, on bronchial epithelial cells revealed transcriptomic signatures indicative of an inflamed, damaged barrier attempting to heal, characterized by increased IL‐33 expression, upregulation of genes related to lipid metabolism, oxidative stress, and cell survival, and downregulation of genes associated with cell adhesion, extracellular matrix, and wound healing [16]. Worryingly, residual active detergents and surfactants can persist in fabrics even after rinsing, potentially impairing epithelial tight junctions (TJs) upon contact [16]. Common surfactants like sodium dodecyl sulfate (SDS) and sodium dodecylbenzene sulfonate, found in countless cleaning products, soaps, shampoos, and cosmetics, have demonstrated the ability to injure lung and skin epithelial TJs even at very high dilutions [14]. This raises concerns about ongoing daily exposure risks.

**Table 2:** Key environmental agents, their categories, common sources, and mechanisms of epithelial barrier disruption.

Category	Substance	Common sources	Primary epithelial target/direct damaging effect	Key cellular pathways affected	Selected key references
Detergents & surfactants	SDS (sodium dodecyl sulfate), anionic surfactants, laundry detergents, dishwasher detergents rinse aids	Household cleaning products (laundry, dishwashing), personal care items (soaps, shampoos, toothpaste)	Disruption of cellular membranes (plasma, mitochondrial, ER), direct damage to tight junctions (TJs) and adherens junctions (AJs), reduced transepithelial electrical resistance (TEER), epithelial cell death	Mitochondrial damage, ROS production, ATP depletion, ER stress, unfolded protein response (UPR), inflammasome activation, cytoskeletal contraction, junctional opening, apoptosis, necrosis, release of alarmins (IL‐33, TSLP), eosinophilic inflammation, potentially ferroptosis	[14, 16, 20, 22, 57, 86]
Food emulsifiers	Polysorbate 20, polysorbate 80, carboxymethyl cellulose, lecithin	Processed foods, food additives.	Increased intestinal permeability, disruption of mucus layer, changes in surface tension, direct cellular toxicity to epithelial cells	Mitochondrial damage via PPAR, β‐oxidation, ROS production, ER stress and UPR, pro‐inflammatory cytokine release, inflammasome activation via ROS, apoptosis	[21, 53, 54, 69]
Micro‐ & nanoplastics	Polystyrene particles	Environmental contamination (water, air, soil), food chain, drinking water, cosmetics	Reduced intestinal mucus secretion; Direct gut barrier damage and increased permeability; epithelial cell penetration; protein binding & conformational changes; lipid bilayer destabilization	Lysosomal damage and enzyme leakage, ROS via NADPH oxidase activation, mitochondrial damage and ROS generation, ER stress (particle accumulation, impaired protein folding), inflammasome activation, ferroptosis, apoptosis, pyroptosis	[19, 58, 59, 60, 95, 111, 131, 150, 151, 254]
Air pollutants	Particulate matter (PM, PM2.5), ozone, diesel exhaust, cigarette smoke components	Combustion processes (traffic, industry), airborne dust, smoke, indoor/outdoor air	Compromised barrier integrity in airway and nasal epithelia, oxidative stress, inflammation, epithelial cell death	Mitochondrial damage and ROS generation, ER stress (PM‐induced Ca^++^ dysregulation), inflammasome activation (NLRP3), ferroptosis (PM), DNA damage, release of alarmins (IL‐33, TSLP) and pro‐inflammatory cytokines (IL‐6, IL‐8, IL‐1β), induction of type 2 immune responses	[17, 18, 62, 83, 96, 110, 114, 130, 152, 244]
Artificial sweeteners	Sucralose, aspartame	Diet foods and beverages, sugar substitutes.	Potential direct interaction with protein structures (sucralose); promotion of amyloid‐like aggregation (aspartame)	ER stress and UPR (impaired protein folding), ferroptosis	[97, 104, 112, 113]

Synthetic detergents were first mass‐produced in the early 20th century, and the steep rise in asthma and AD paralleled the household uptake of sulfated anionic surfactants during the 1960s [49]. Patch testing with SLS causes a reproducible increase in transepidermal water loss and a fall in stratum corneum hydration, confirming direct cutaneous barrier compromise [50, 51]. Professional cleaners chronically exposed to detergents show higher transepithelial water loss and a greater risk of work‐related hand dermatitis, which links occupational contact to sustained barrier injury [52]. Even diluted laundry detergent aerosolized from treated fabrics upregulates IL25, IL33, and thymic stromal lymphopoietin (TSLP) in airway epithelium, illustrating how trace residues can initiate epithelitis and prime type 2 immune pathways [16].

The widespread use of emulsifiers in the food industry is increasingly scrutinized as a potential contributor to gut barrier‐related diseases. Processed food emulsifiers have been shown to increase intestinal permeability, potentially via mechanisms involving surface tension changes and direct cellular toxicity, similar in some ways to detergents. Emulsifiers, such as polysorbates, lecithin, carboxymethylcellulose, and sorbitolmonostearate, thicken the mucosal surface fluid, trap commensal bacteria, disrupt mucus–bacterial interactions, and drive colitis‐like inflammation in mice [53, 54]. Even low concentrations can lead to mucosal damage, as observed in animal models [55]. Emulsifier exposure may also trigger epithelial cell activation and facilitate bacterial translocation across the compromised barrier [21, 54, 56]. A recent study showed that exposure to food emulsifiers polysorbate 20 and 80, even at concentrations 20 times more diluted than the allowed amount, led to cellular stress and pro‐inflammatory responses in gut epithelial cells [21]. Residual rinse aid and dishwasher detergent left on ‘clean’ crockery are being investigated as a previously overlooked source of esophageal and intestinal barrier injury [20, 57].

Micro‐ and nanoplastics represent an emerging skin and mucosal barrier toxin class: polystyrene microplastics reduced intestinal mucus secretion and damaged gut barrier function in mice, while 1 μm particles penetrated the epithelium more readily than larger fragments [19, 58]. At the nanoscale, plastic particles bind proteins, distort secondary structure, destabilize lipid bilayers, and activate pro‐inflammatory transcription programs, culminating in oxidative stress‐driven epithelial cell death [59, 60].

**Fig. 2:**
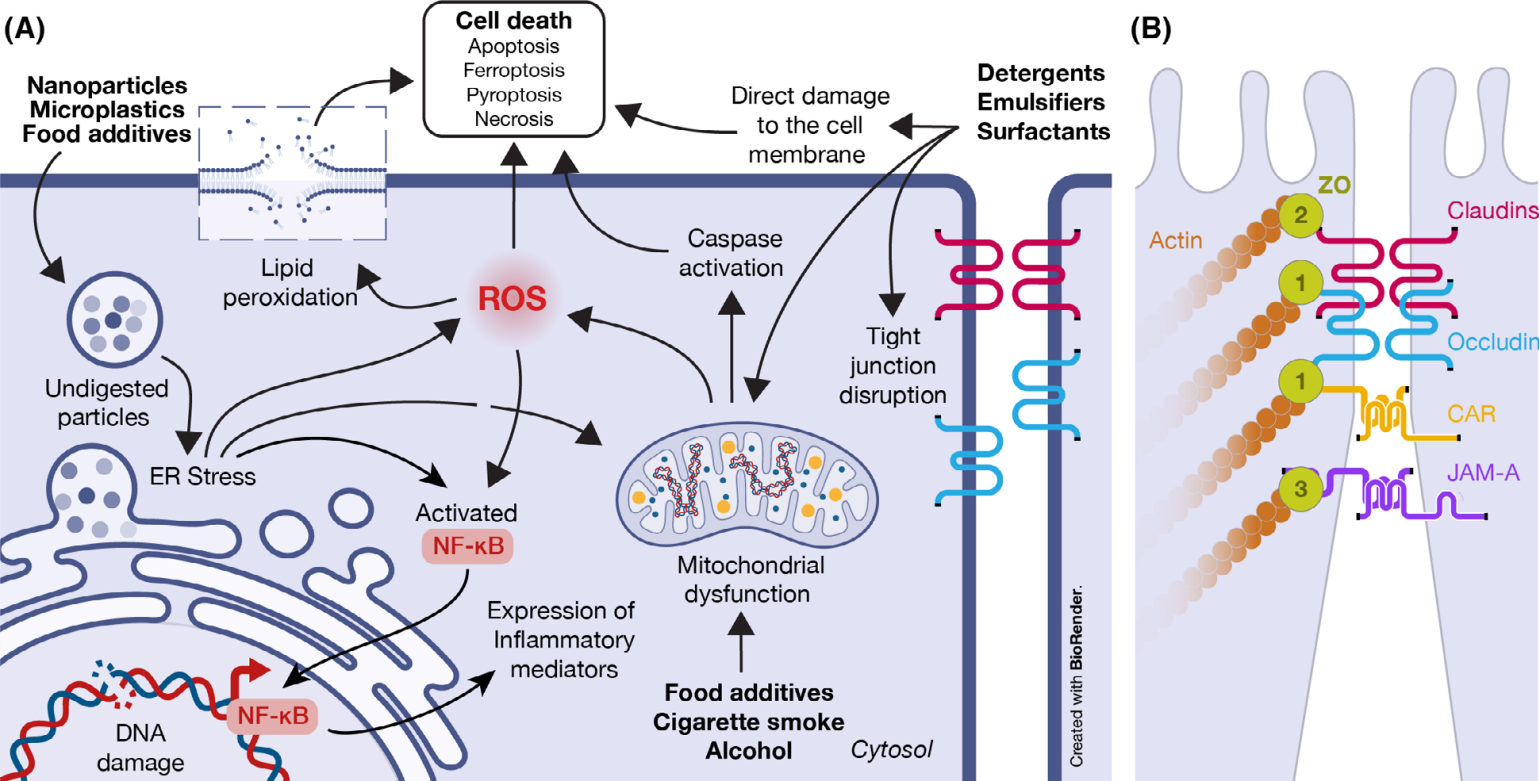
(A) Mechanisms by which environmental toxicants induce cellular damage. Undigested particles (micro‐ and nanoplastics) trigger endoplasmic reticulum (ER) stress and oxidative stress, leading to NF‐κB activation. Alcohol and cigarette smoke cause mitochondrial dysfunction, resulting in reactive oxygen species (ROS) generation and caspase‐mediated apoptosis. Surfactants (detergents and emulsifiers) primarily disrupt cellular membranes, causing electrolyte leakage, mitochondrial impairment, tight‐junction disruption, and additional ROS production. Excess ROS leads to DNA strand breaks, the release of damage‐associated molecular patterns (DAMPs) and NF‐κΒ signaling. If these insults are not resolved, they culminate in multiple forms of cell death—including apoptosis, pyroptosis, ferroptosis, and necrosis. (B) Schematic view of the principal tight‐junction architecture showing barrier‐forming and pore‐forming claudins, tight junction‐associated MARVEL proteins (TAMPs), junctional adhesion molecules (JAMs), the coxsackie and adenovirus receptor (CAR), zonula occludens‐1 (ZO‐1), and their linkage to the Actin‐myosin cytoskeleton.

As Paracelsus said 500 years ago, ‘Sola dosis facit venenum’, ‘Only the dose makes the poison’. Studies have shown that the dose and time of exposure to these substances are critical in determining their cellular and molecular toxicity [20, 21, 22]. The molecular mechanisms of how environmental toxic substances cause cellular damage, the discovery of which is key to being able to mitigate their toxic effects, have been started to be elucidated; they include multiple intertwined pathways, explained below, that lead to dysregulation in cellular metabolism, cellular stress, and cell death [18, 22, 57, 61, 62]. Although environmental toxic substances may initiate the cascade of cellular stress from different points, as most intracellular stress mechanisms converge, they tend to lead to similar toxic effects in epithelial cells (Fig. 2A). Many of the subsequent mechanistic details are based on *in vitro* studies; while these are essential for understanding cellular processes, their extrapolation to *in vivo* physiology should be approached with due consideration.

## Intracellular pathways of epithelial cell toxicity

### ROS as a central mediator in toxic substance‐induced cellular stress

Environmental toxic substances trigger a cascade of interconnected intracellular stress events, with reactive oxygen species (ROS) often emerging as a common and pivotal mediator. While the initial points of impact may vary depending on the toxicant's chemical structure, the resulting oxidative stress, mitochondrial dysfunction, and endoplasmic reticulum (ER) stress are frequently interlinked, culminating in cellular damage and epithelial barrier compromise.

Mitochondrial damage and the dysregulation of the electron transport chain represent a central process leading to both ROS generation and the disruption of key metabolic processes, such as oxidative phosphorylation (OXPHOS) [63, 64, 65]. How environmental toxic substances cause mitochondrial damage and loss of mitochondrial membrane potential depends on the substance's chemical structure. For example, SDS or SLS, a surfactant, disrupts cellular membranes, including mitochondrial membranes [66]. Damage to the inner mitochondrial membrane may also cause leakage of electrons into the matrix, generating $O_{2}^{-}$ radicals and initiating a cascade of ROS generation [63, 67]. Another example is the food emulsifier polysorbate 80 (P80), which structurally contains an oleic acid molecule bound to a sugar moiety [68]. P80 can lead to mitochondrial membrane damage and ROS generation, as well as activate peroxisome‐proliferator‐activated receptor and cause an increase in β‐oxidation, resulting in excess ROS formation [21, 69]. Mitochondrial damage not only leads to ROS generation but also results in dysregulation of cellular metabolism, depleting the OXPHOS capacity and diminishing the ATP generation capacity of the cell [64, 70, 71]. This is followed by downstream effects, including disrupted protein folding [72], and decreased capacity of the cell to mitigate various stress factors, such as further oxidative stress [64, 73], ER stress [72], genotoxic stress [74, 75], and calcium imbalance [76, 77, 78]. Furthermore, severe damage to the mitochondrial membrane can lead to the leakage of cytochrome c into the cytosol, potentially initiating apoptosis [79, 80].

The surge in ROS from dysfunctional mitochondria or from other cellular compartments, such as the plasma membrane/endosome/lysosomes (via NADPH oxidase, particularly activated by phagocytosed, nondegradable particles like particulate matter and micro‐nanoplastics that resist lysosomal degradation [81, 82, 83]), peroxisomes, or the ER itself during stress responses [81, 84, 85], becomes a common pathogenic hub [18, 20, 69, 86, 87, 88]. This ROS can directly attack cellular macromolecules and trigger specific damage pathways. A critical consequence is extensive membrane lipid peroxidation. This process, involving enzymatic lipid peroxidation through lipoxygenases and nonenzymatic lipid peroxidation via Fenton reactions with iron, can be initiated by environmental toxic substances containing lipid‐like structures with double bonds (e.g., most food emulsifiers like P80 and soy lecithin) [89, 90, 91], and can overwhelm cellular antioxidant capacities, leading to ferroptosis—a form of cell death dependent on iron and characterized by excessive lipid peroxidation [92, 93, 94]. Substances that trigger excessive ROS generation, such as micro‐nanoplastics, particulate matter, and non‐nutritional sweeteners like sucralose and aspartame, have been shown to induce ferroptosis [95, 96, 97]. While food emulsifiers and SDS have not been shown to induce ferroptosis directly, it is probable that they also trigger this pathway through ROS‐mediated lipid peroxidation [20, 57]. These events cause changes in membrane stiffness, fluidity, and permeability, ultimately resulting in plasmalemma rupture and cell death [98].

In addition to direct oxidative damage to lipids, ROS profoundly impacts protein homeostasis and ER function. The controlled formation of disulfide bonds is critical for the proper folding of proteins in the ER lumen [99, 100]. Excess ROS in the ER lumen, whether originating from damaged mitochondria or other sources, may lead to the premature and incorrect formation of these bonds, preventing proteins from achieving their intended tertiary structure [101, 102]. This accumulation of misfolded proteins triggers the unfolded protein response (UPR), a physiological mechanism designed to restore ER homeostasis [99, 100]. However, if the toxic stimulus is overwhelming or persistent, the UPR becomes maladaptive, initially promoting autophagy and, if still unresolved, ultimately leading to inflammatory signaling and cell death, primarily through apoptosis [103]. Some environmental toxic substances, such as aspartame, may initially cause protein folding defects that lead to an excess UPR, which in turn causes increased ROS inside the ER lumen; if this exceeds the ER's antioxidant capacity, it can disrupt membranes and leak out [104, 105, 106, 107]. Besides ROS‐induced misfolding, other environmental toxicants can directly compromise the ER: surfactants like SDS can disrupt the ER membrane, similar to other cellular membranes, causing dysfunction of membrane‐associated proteins and disturbing Ca^++^ and ROS homeostasis in the ER lumen [66, 76, 108, 109]. Particulate matter has been shown to disrupt Ca^++^ homeostasis within the ER lumen by interacting with membrane proteins, resulting in ER stress and apoptosis [110]. Microplastics have also been documented to accumulate within the ER lumen, impairing protein folding and causing ER stress in human syncytiotrophoblasts [111]. Artificial sweeteners, such as sucralose and aspartame, also induce ER stress; sucralose may interact with hydrophobic protein surfaces [112], and aspartame can lead to the self‐assembly of amyloid‐like structures [113], suggesting that protein aggregation may also hinder proper folding. While the precise initiating mechanisms through which many environmental toxic substances cause a sustained UPR remain to be fully elucidated, their ROS‐generating capabilities and direct interactions with membranes and proteins represent plausible links [21, 104, 114, 115].

Ultimately, whether originating from mitochondrial dysfunction, direct membrane damage, or ER stress, supraphysiologic levels of ROS can act as signaling molecules. Two transcription factors commonly activated are NF‐κB and AP‐1, which orchestrate the release of pro‐inflammatory cytokines and mediators, propagating stress signals to surrounding cells and contributing to tissue inflammation [116, 117, 118, 119]. The direct attack by ROS on major cellular building blocks—amino acids, lipids (which also causes Ca^++^ leakage into the cell [120], disrupting intracellular signaling pathways and activating Ca^++^‐dependent proteases), and nucleic acids—further compounds the damage, leading to dysfunctional cells and, consequently, a dysfunctional epithelial barrier [85, 90, 91, 121, 122].

### Inflammasome & pyroptosis

Pyroptosis is a form of regulated cell death that is canonically caused by inflammasome activation, leading to the release of IL‐1β and IL‐18, as well as the cleavage of Gasdermin D. This process results in water influx, cellular swelling, and cell death [123, 124, 125]. Unlike apoptotic cell death, pyroptosis leads to the release of cytokines and cellular contents, which act as damage‐associated molecular patterns (DAMP)s to trigger and perpetuate inflammation [123, 126]. Numerous triggers exist for NLRP3 inflammasome activation, including K^+^ efflux, lysosomal damage, ROS generation, and bacterial cell wall components [127, 128]. Other inflammasomes, such as NLRC4, AIM2, NLRP1, and Pyrin, are primarily activated by microbial products, including cell wall components, dsDNA, or toxins [127, 128]. AIM2 can be activated through the binding of dsDNA, regardless of its source, whether derived from bacterial, host‐nuclear, or host‐mitochondrial origins [129].

The initial insult from various environmental toxic substances can lead to inflammasome activation in epithelia through multiple mechanisms. Micro‐nanoplastics and particulate matter result in lysosomal damage and ROS generation, resulting in inflammasome activation and pyroptotic cell death [95, 96, 130, 131]. Membrane‐disrupting substances, such as SLS disrupt the fluidity and permeability of cellular membranes and cause disturbances in ion homeostasis, which may result in K^+^ leakage and inflammasome activation. Substances, such as p80 or SLS, may also cause inflammasome activation through ROS generation and mitochondrial damage [21, 132, 133].

Environmental toxic substances not only damage the epithelia directly but also cause microbial dysbiosis [54, 134, 135, 136]. The presence of dysbiotic bacteria causes further damage to the epithelia and can lead to increased inflammasome activation through engulfment by epithelia and intracellular release of components or toxin production [137, 138]. As the integrity of the epithelial barrier is compromised, both commensal and pathogenic bacteria start dislocating to the lamina propria [1]. This results in excess activation of macrophages encountering these bacteria and further inflammasome activation. Environmental toxic substance‐induced epithelial injury and microbial dysbiosis synergistically undermine barrier integrity, promoting bacterial translocation and a self‐amplifying cycle of inflammasome‐driven inflammation [139].

Environmental toxins provoke epithelial inflammasome activation and pyroptosis through mechanisms like lysosomal damage, ROS, and ion flux, while external stresses drive microbial dysbiosis that further compromises barrier integrity. This dual assault facilitates bacterial translocation and perpetuates a vicious cycle of inflammasome‐driven inflammation and epithelial barrier injury.

### DNA damage/p53 axis and apoptosis

We have mentioned above multiple mechanisms of cellular stress leading to cellular dysfunction when cells encounter environmental toxic substances. If these stressors persist, they can overwhelm cellular defense mechanisms, leading to cell death. There are several mechanisms of cell death, including programmed cell death, such as apoptosis [20, 21, 140], ferroptosis [90], and pyroptosis [132, 141, 142], as well as nonprogrammed cell death, that is, necrosis, which can occur when the cell's defense mechanisms are overcome due to constant or high‐dose exposure to ETS.

Apoptosis is a tightly regulated mechanism of cell death triggered by intrinsic and extrinsic factors [80]. Intrinsic triggers include nutrient and growth factor deprivation, DNA damage, ER stress, oxidative stress, mitochondrial membrane damage, and Ca^++^ overload [143, 144, 145]. As mentioned previously, nearly every trigger of the intrinsic apoptotic pathway can be initiated by environmental toxic substance exposure.

DNA damage is sensed by p53 by activation of ATM/ATR kinases [146], which induces cell cycle arrest via the induction of p21, an inhibitor of the Cdk2/Cyclin E complex, enforcing a G_1_/S checkpoint arrest [147]. This provides an opportunity for DNA repair. If the damage is too extensive or the repair mechanisms are overwhelmed, p53 triggers the production of pro‐apoptotic proteins, namely Noxa, BID, and Puma, leading to the initiation of apoptosis [148]. Increased DNA breaks from excess ROS generation due to persistent or high‐dose environmental toxic substance exposure can be a major contributor to apoptotic cell death.

Unresolved ER stress causes sustained UPR activation, which can lead to pro‐apoptotic signals through multiple pathways of ER stress. PERK–eIF2α signaling drives ATF4‐mediated CHOP induction, which downregulates Bcl‐2 and upregulates BH3‐only proteins (e.g., Bim), tipping the mitochondrial permeabilization balance [100, 149]. Additionally, ER Ca^2+^ leakage activates calpains and ER‐resident caspase‐12, linking ER stress to the intrinsic caspase cascade [149]. As most environmental toxic substances result in chronic ER stress, the downstream signals of the UPR signaling pathway can be another contributor to environmental toxic substance‐induced apoptotic cell death.

Mitochondrial damage that leads to dysfunction and dysregulation is a central aspect of environmental toxic substance‐induced damage in cells. As mentioned, this can occur through direct damage to the mitochondrial membrane by surfactants like SDS [66] or through indirect membrane damage caused by excess ROS generation and lipid peroxidation from multiple substances, such as micro‐nanoplastics [150, 151], particulate matter [152], and SDS. Damage to the mitochondrial membrane can lead to apoptosis via the leakage of cytochrome c and the second mitochondria‐derived activator of caspases (SMAC) into the cytoplasm [153]. Cytochrome c associates with APAF‐1/Casp9, while SMAC prevents its inactivation, thus triggering the apoptotic cascade, providing another route through which environmental toxic substances can induce apoptosis.

It is well known that excess ROS is a major cause of increased double‐strand (ds) DNA breaks [154, 155, 156]. Excess ROS caused by exposure to environmental toxic substances leads to dsDNA breaks. Although DNA break repair is a robust process in epithelial cells, chronic exposure to these substances at low doses may result in ongoing dsDNA break‐repair cycles, ultimately leading to mutations, as seen in chronic inflammation, which is a well‐established risk factor for the development of malignancies [157, 158].

Environmental pollutants may drive cells toward demise through DNA damage, protein‐folding machinery overload, oxidative stress provocation, and mitochondrial integrity compromise. Chronic exposure, therefore, leads to excessive cell loss as well as tissue injury [1]. This underscores the importance of reducing pollutants and improving cellular defenses.

### Cytoskeletal contraction and early junctional opening

**Fig. 3:**
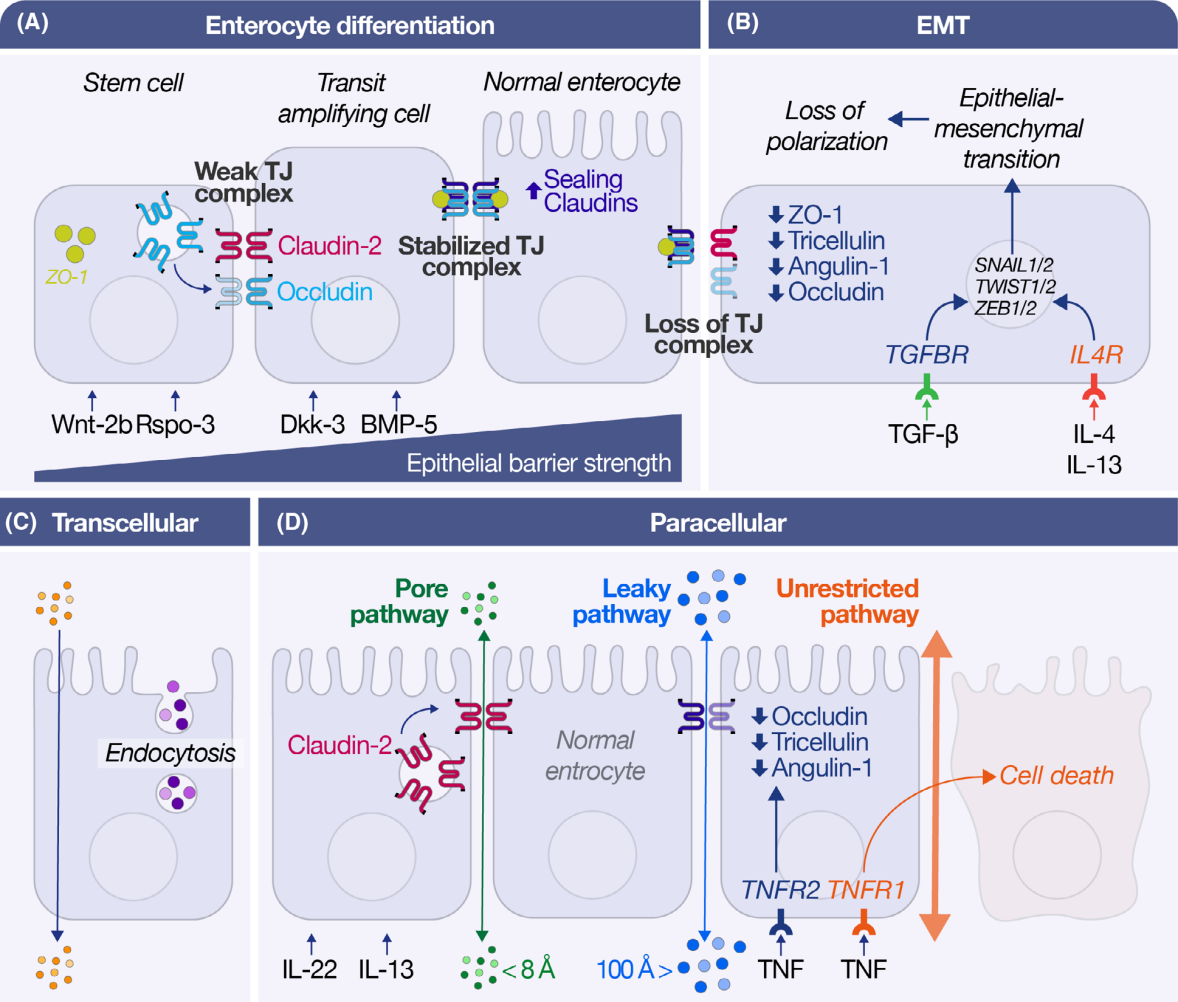
(A) Wnt‐2b and R‐spondin‐3 sustain stem‐cell proliferation, whereas Dkk‐3 and BMP‐5 drive maturation; weak claudin‐2‐rich junctions in transit‐amplifying cells are gradually replaced by sealing claudins and reinforced ZO‐1 strands, increasing epithelial barrier strength. (B) TGF‐β (via TGFBR) or IL‐4/IL‐13 (via IL4R) initiate the epithelial‐to‐mesenchymal transition, downregulating ZO‐1, tricellulin, angulin‐1, and occludin, while inducing SNAIL, TWIST, and ZEB transcription factors and erasing apicobasal polarity. (C) Vesicular transcytosis ferries luminal macromolecules across enterocytes, providing a tight‐junction–independent, size‐flexible transport route. (D) Paracellular permeability is shaped by three TNF‐responsive conduits: a claudin‐2 pore pathway (< 8 Å) amplified by IL‐22 or IL‐13; a leaky pathway (~ 100 Å) created by loss of occludin, tricellulin, and angulin‐1; and an unrestricted pathway that arises when TNF‐induced epithelial cell death leaves transient gaps between neighboring cells.

All major barrier epithelia—from the gut and airway to the skin and esophagus—can undergo a rapid increase in paracellular permeability due to cytoskeletal contractions. Under homeostasis, TJ and adherens junction (AJ) complexes seal the space between epithelial cells and are tethered to the peri‐junctional actin‐myosin ring (Fig. 2B, Table 3). The inherent resilience and baseline integrity of these barriers are crucial; they are established and maintained through cellular differentiation processes that progressively mature intercellular junctions and enhance overall barrier strength, involving specific molecular regulators and changes in junctional protein composition (Fig. 3A). Early in inflammation or injury, various triggers (pro‐inflammatory cytokines like TNF‐α or IL‐13, ROS from oxidative stress, surges in intracellular Ca^++^ levels, and environmental toxicants) initiate signaling events that target this actomyosin apparatus [159]. These stimuli cause the circumferential actomyosin belt to contract, exerting tension on cell–cell contacts and pulling junctions slightly apart. The result is an ‘early junctional opening’, where small gaps form between cells, and transepithelial resistance drops before any widespread cell death or gross tissue damage occurs. Notably, this early, transient barrier defect is often reversible if the cytoskeletal tension is released or counteracted, but it can also progress to larger junctional disassembly if the stimuli persist.

**Table 3:** Key tight‐junction proteins and their predominant expression/role in various epithelial tissues.

Protein family	Specific protein(s)	General function(s) in tight junctions	Predominant epithelial tissue expression/key roles
Claudins	Claudin‐1	Barrier‐forming; seals paracellular space; maintains cell polarity; epidermal barrier integrity	Skin (epidermis): Essential for water barrier function Intestine: Contributes to barrier properties Lung: Present in airway epithelium Kidney: Expressed in various nephron segments
	Claudin‐2	Pore‐forming (cation‐selective, primarily Na^+^, Ca^2+^); increases paracellular water flux	Intestine (small & large): Contributes to Na^+^ and water reabsorption; upregulated in IBD (e.g., UC by IL‐13) Kidney (proximal tubule): Involved in Na^+^ reabsorption Liver (bile ducts): Regulates bile composition
	Claudin‐3, Claudin‐4	Barrier‐forming; contribute to epithelial tightness. Claudin‐4 can act as a receptor for CPE	Various epithelia: Widely expressed, including intestine, lung, kidney, pancreas, breast Claudin‐4 in intestine: Contributes to barrier; target for Clostridium perfringens enterotoxin (CPE) Skin: Present
	Claudin‐5	Barrier‐forming; predominantly in endothelial tight junctions, but also some epithelia	Endothelium (blood–brain barrier, blood‐retinal barrier): Critical for vascular integrity Lung epithelium (alveoli): Contributes to alveolar barrier Kidney (glomerulus): Expressed in podocytes
	Claudin‐7	Barrier‐forming; ion transport regulation; cell adhesion	Intestine (colon): Important for barrier function; mislocalization in IBD Kidney (collecting duct): Regulates paracellular Cl^−^ transport Skin: Present
	Claudin‐10 (a & b)	Pore‐forming (Claudin‐10a: anion‐selective; Claudin‐10b: cation‐selective)	Kidney: Different isoforms in distinct nephron segments regulating ion transport Skin (sweat glands): CLDN10B crucial for sweat ion composition (mutations cause HELIX syndrome) Intestine: Expressed
	Claudin‐15	Pore‐forming (cation‐selective, Na^+^); contributes to fluid and nutrient absorption	Intestine (small intestine): Forms Na^+^ pores, crucial for nutrient and water absorption
	Claudin‐16 (Paracellin‐1)	Pore‐forming (Mg^++^ and Ca^++^ selective channel in complex with Claudin‐19)	Kidney (thick ascending limb): Essential for paracellular reabsorption of Mg^++^ and Ca^++^. Mutations cause familial hypomagnesemia with hypercalciuria and nephrocalcinosis (FHHNC)
Occludin	Occludin	Barrier‐forming; regulates paracellular permeability; scaffolding & signaling roles; cell polarity	Most Epithelia & endothelia: A key component of TJs (intestine, lung, kidney, skin, BBB). Target for disruption by pathogens/cytokines (e.g., Der p1, TNF‐α)
Tricellular TJ proteins	Tricellulin (MARVELD2)	Seals tricellular contacts; prevents macromolecule leakage at three‐cell junctions	Various epithelia: Concentrated at tricellular TJs (intestine, kidney, inner ear). Reduced expression in UC
	Angulins (ILDR1/2, LSR)	Recruit tricellulin to tricellular TJs; essential for tricellular barrier function	Various epithelia: Co‐localize with tricellulin at tricellular TJs
JAMs (junctional adhesion molecules)	JAM‐A (F11R), JAM‐B, JAM‐C	Cell–cell adhesion; leukocyte transmigration; scaffolding; TJ assembly & maintenance	Epithelia & endothelia: Widely expressed (intestine, lung, kidney). JAM‐A is involved in barrier regulation and immune cell interactions
CAR (coxsackie and adenovirus receptor)	CAR	Viral receptor; cell adhesion; TJ localization and barrier function	Various Epithelia: Brain, heart, pancreas, intestine, airway. Contributes to TJ's integrity
ZO (zonula occludens) proteins	ZO‐1, ZO‐2, ZO‐3	Scaffolding proteins (MAGUK family); link TJ transmembrane proteins (claudins, occludin, JAMs) to the actin cytoskeleton; signaling roles; regulate TJ assembly and function	All TJ‐forming cells: Ubiquitous plaque proteins essential for TJ structure and function. ZO‐1 is a key marker for TJs. Disrupted/mislocalized in many barrier diseases (e.g., IBD, asthma, and AD)

Mechanistically, cytoskeletal contraction at the apical junction is driven by classical Rho GTPase and Ca^++^‐dependent pathways. RhoA activation and its effector Rho‐associated kinase (ROCK) stimulate myosin II activity by phosphorylating the myosin light chains (either directly or via intermediate kinases), while Ca^++^‐calmodulin signaling activates myosin light‐chain kinase (MLCK) with a similar outcome [159]. Phosphorylation of myosin II regulatory light chains triggers contraction of the peri‐junctional actin ring, which increases tension on TJs and AJs and can force open the TJ strands. In epithelial cells exposed to inflammatory cytokines like TNF‐α or IL‐1β, for example, MLCK is activated and contributes to barrier loss [159]. This causes modest tightening of the junctional belt and a reversible increase in permeability, often without immediate disruption of the junction structure. By contrast, strong activation of RhoA–ROCK (for instance, via Rho GEF‐H1 released from microtubules during stress) induces more pronounced actomyosin contraction and can drive actual junctional disassembly [159]. Consistently, experimental models show that sustained MLCK activity alone causes only a minor (~ 20%) drop in epithelial resistance with intact TJs, whereas excessive RhoA signaling leads to dramatic AJ/TJ disruption [159]. In parallel with force generation, contractile signaling can also provoke remodeling of junction proteins—for instance, endocytosis of TJ components like occludin is triggered by MLCK‐dependent actomyosin pulls during TNF‐α exposure, weakening the seal [159]. Thus, the early phase of barrier opening involves a concerted mechanism: phosphorylation‐driven myosin contraction that physically enlarges the paracellular space, accompanied by dynamic changes in junctional protein localization.

Beyond these canonical pathways, a variety of cytoskeletal‐ and junction‐associated regulators fine‐tune actomyosin contraction and junctional opening. The actin‐binding scaffold cortactin is one such regulator; it co‐localizes with ZO‐1 at TJs and appears to restrain excessive contractility, as cortactin deficiency leads to heightened RhoA–ROCK activity and barrier leakage in intestinal epithelia (with loss of ZO‐1 and E‐cadherin at contacts) [160]. On the other hand, the mechanosensitive protein vinculin (recruited by α‐catenin at AJs) is required to stabilize junctions under stress. Upon increased tension, vinculin is rapidly recruited to junctional sites—especially tricellular junctions—where it anchors cortical actin and myosin II to the membrane; without vinculin, actin disengages from the junction, and TJ proteins like ZO‐1 show breakage, resulting in local leaks [161]. Tight‐junction plaque proteins, such as cingulin (and its paralog paracingulin), also link the contractile apparatus to junctions and modulate Rho GTPase signaling, effectively tethering myosin II filaments to the TJ complex [159]. Similarly, the submembrane spectrin–adducin network has emerged as a structural support that connects cortical actin to junctional complexes, acting as a mechanical transducer to maintain adhesion during active remodeling [162]. Additional cytoskeletal linkers like the ERM family (ezrin, radixin, and moesin) contribute by anchoring actin to apical membrane proteins, thereby organizing microvilli and the apical cortex in ways that support junction stability [163]. Signaling molecules also play nuanced roles in early junctional regulation: for example, Ca^++^/calmodulin‐dependent protein kinase II (CaMKII) has been identified as a negative regulator of TJ assembly (its inhibition actually strengthens the barrier) [164], and LIM domain kinases (downstream of Rho) phosphorylate cofilin to stabilize F‐actin—a change that can increase junctional tension and permeability [159]. Through their structural and signaling interactions with the actin cytoskeleton, these regulators (cortactin, vinculin, cingulin, spectrin/ERM networks, CaMKII, LIMK, and others) integrate with the classic Rho–myosin pathways to determine the extent and reversibility of junction opening.

These early cytoskeletal and junctional changes have important disease ramifications across different organs. In the gut, excessive cytokine‐induced contraction of the peri‐junctional actin ring underlies the ‘leaky gut’ phenomenon in IBD. For instance, during Crohn's disease or ulcerative colitis (UC), immune‐derived TNF‐α and interferon‐γ drive MLCK‐mediated myosin phosphorylation and occludin removal from TJs, compromising intestinal barrier function [159]. This permits antigen and microbe translocation that can exacerbate mucosal inflammation. In the airway epithelium of asthmatic patients, Th2 cytokines, such as IL‐4 and IL‐13, similarly induce early junctional opening. Experiments show that IL‐13 exposure causes a significant drop in transepithelial electrical resistance (TEER) and increases macromolecular flux across bronchial cell layers, accompanied by redistribution or loss of junction proteins like ZO‐1 and E‐cadherin [165]. Environmental pollutants (e.g. ozone and particulate matter) and allergens can amplify this by causing oxidative stress and Ca^++^ influx in airway epithelia, further activating Rho/MLCK pathways and contributing to the barrier dysfunction in asthma. In AD (eczema), the skin's barrier defect is partly due to cytoskeletal‐junctional alterations: chronic Th2 inflammation (high IL‐4/IL‐13) downregulates key junction proteins (like claudin‐1) and loosens TJs, while repeated scratching triggers Ca^++^‐dependent actomyosin contraction in epidermal cells. The result is increased epidermal permeability (reflected by lower electrical resistance in lesional skin) and greater entry of allergens and microbes, feeding a vicious cycle of inflammation [9]. Likewise, in EoE, an allergic inflammatory condition of the esophagus, elevated IL‐13 levels lead to an early breakdown of the epithelial barrier. IL‐13 not only alters junctional protein expression but also induces an epithelial protease (calpain‐14) that cleaves cytoskeletal or junctional components, thereby weakening cell–cell adhesion [166]. This makes the esophageal epithelium leaky to dietary antigens and irritants, provoking chronic eosinophilic inflammation. Across these disorders, the common theme is that diverse stimuli initiate actomyosin‐driven junctional opening as an early event in pathogenesis. Therefore, targeting the regulatory nodes of cytoskeletal contraction (e.g., using ROCK inhibitors or cytokine blockers) is being explored as a strategy to reinforce epithelial barriers and mitigate disease severity [160, 165].

### Epithelial–mesenchymal transition and peri‐epithelial fibrosis

Epithelial‐to‐mesenchymal transition (EMT) is a process whereby epithelial cells lose polarity and adhesion, becoming mesenchymal‐like and motile (Fig. 3B). Across barrier tissues (gut, skin, airways, esophagus), chronic insults can trigger EMT, driving an early ‘leaky’ epithelium toward long‐term structural remodeling [167, 168]. Under persistent inflammation, epithelial TJs and AJs are disrupted, compromising the barrier and allowing pathogens and antigens to penetrate. Simultaneously, epithelial cells begin to assume mesenchymal characteristics, contributing to tissue fibrosis and architectural changes. This phenomenon has been observed in disorders ranging from IBD to asthma, in which the epithelium orchestrates fibrotic remodeling via EMT in response to sustained injury [167, 168]. Overall, EMT serves as the bridge between epithelial barrier dysfunction and the permanent remodeling that underlies chronic inflammatory diseases [167, 168].

Fibrosis results from the mesenchymal phenotype obtained by cells after undergoing EMT at damaged epithelial barriers. A good example of this is *lamina reticularis* thickening beneath the basal lamina in asthmatic bronchi, which is readily observed at the time of first diagnosis, as shown in biopsies taken on the day of initial diagnosis of asthma in children [5]. We presume that this second layer of barrier develops in epithelial barrier leaky individuals to protect the deeper tissues from environmental pollutants, the microbiome, allergens, and toxins.

Persistent inflammation in barrier tissues provides a rich milieu of cytokines and growth factors that induce EMT. Transforming growth factor‐β1 (TGF‐β1) is the prototypical EMT driver, and it is often elevated in chronic inflammation. In addition, type 2 helper T‐cell (Th2) cytokines like IL‐4 and IL‐13, as well as Th17 cytokines like IL‐17A, synergize with TGF‐β1 to downregulate epithelial junctional proteins and upregulate mesenchymal genes [168, 169]. For example, AD lesions (a Th2‐dominated inflammation) show increased Snail1 and Twist transcription factors in keratinocytes, along with co‐expression of epithelial keratins and mesenchymal vimentin—evidence of partial EMT in the skin [169]. Similarly, in severe asthma, IL‐13 and IL‐17 levels are high; these cytokines induce airway epithelial cells to lose E‐cadherin and gain α‐smooth muscle actin (α‐SMA), indicating an EMT program [168]. The classic pro‐inflammatory cytokines tumor necrosis factor‐α (TNF‐α) and IL‐1β also contribute by activating NF‐κB and other pathways that promote EMT. Indeed, long‐term exposure to TNF‐α and IL‐1β can drive otherwise normal epithelial cells to acquire mesenchymal traits (e.g., vimentin and fibroblast‐specific protein) in culture and in wounds [170, 171]. In summary, chronic exposure to cytokines, such as TGF‐β1, IL‐13/IL‐4, IL‐17, TNF‐α, and IL‐1β, creates a pro‐EMT microenvironment that makes epithelial barriers leaky and primes and stimulates them for structural change to develop a secondary barrier.

Beyond cytokines, a variety of environmental toxicants and irritants can directly injure epithelial barriers and initiate EMT. In the gut, ingested environmental microparticles are significant EMT triggers. Microplastics—tiny plastic particles now ubiquitous in food and water—have been shown to disrupt the intestinal barrier, causing a ‘leaky gut’. Chronic oral microplastic exposure leads to microbiome dysbiosis, epithelial TJ loss, and mucosal immune activation. As a result, pro‐inflammatory cytokines and bacterial toxins translocate across the gut wall, fueling further inflammation and tissue injury. Processed food additives like emulsifiers (e.g., polysorbate 80) similarly act as detergents in the gut lumen. At sub‐toxic concentrations, these emulsifiers can strip away mucus and disturb epithelial TJs, lowering transepithelial resistance and provoking a pro‐inflammatory transcriptomic response in intestinal cells [21]. Airborne pollutants are another EMT trigger: particulate matter (such as diesel exhaust, smoke, or carbon nanoparticles) chronically inhaled into airways injures the respiratory epithelium and triggers repair responses. Studies show that exposure to cigarette smoke or fine particulates can induce EMT in bronchial epithelial cells—especially in combination with inflammatory cytokines like IL‐17—leading to loss of E‐cadherin and gain of mesenchymal markers in the airway lining [168]. Thus, environmental toxins (from microplastics to air pollution) can initiate epithelial stress and inflammation that set off EMT programs, compounding barrier dysfunction [1, 168].

At the cellular level, inflammation‐ or toxin‐induced EMT is driven by well‐characterized molecular mechanisms. Key EMT‐activating transcription factors—including SNAIL1/2 (Snail and Slug), TWIST1/2, and ZEB1/2—are upregulated in epithelial cells under EMT‐inducing conditions [168]. These transcription factors repress epithelial genes (such as CDH1 encoding E‐cadherin) and activate mesenchymal genes, effectuating a ‘cadherin switch’ (downregulating E‐cadherin and upregulating N‐cadherin) and intermediate filament shift (increasing vimentin) [168]. The loss of E‐cadherin, along with downregulation of TJ proteins like claudins and occludin, leads to the dissolution of AJs, TJs, and desmosomes between cells [168]. Consequently, epithelial cells lose their polarity and cell–cell contacts, becoming spindle‐shaped and migratory. Cytoskeletal remodeling is a hallmark of this transition: EMT stimulates actin stress fiber formation and focal adhesions, often through Rho GTPase signaling, which enhances cell contractility and motility. EMT also endows epithelial‐derived cells with invasive capabilities by inducing matrix‐degrading enzymes. Notably, EMT‐activated cells begin secreting matrix metalloproteinases (MMPs), such as MMP‐2 and MMP‐9, that degrade the basement membrane and local extracellular matrix [168]. Degradation of the underlying basement membrane is a crucial step in allowing epithelial cells to detach and migrate into the tissue as mesenchymal (fibroblast‐like) cells. In parallel, EMT cells upregulate extracellular matrix components (fibronectin, collagen I, etc.) and α‐SMA, adopting a myofibroblast‐like phenotype [168]. This myofibroblastic transformation enables formerly epithelial cells to contribute to fibrosis by depositing collagen and contracting the matrix. In summary, EMT involves coordinated reprogramming: master transcription factors silence epithelial adhesion molecules (like E‐cadherin) and induce mesenchymal genes (vimentin, N‐cadherin, and α‐SMA), causing junctional disassembly, cytoskeletal reorganization, MMP activation, and basement membrane breakdown [168]. These molecular events convert a stable barrier epithelium into a migratory, matrix‐remodeling cell population at the core of tissue structural changes.

EMT has significant pathological consequences for barrier tissues, linking chronic inflammation to disease progression. As epithelial cells undergo EMT and lose their cohesive barrier function, the tissue becomes more permeable—essentially a barrier ‘failure’. A leaky barrier permits increased infiltration of antigens, microbes, and environmental particles into the tissue, which in turn recruits immune cells and amplifies local inflammation in a vicious cycle. In IBD, for example, long‐standing intestinal inflammation triggers epithelial EMT, and these EMT‐derived mesenchymal cells directly contribute to fibrosis (excessive ECM deposition) in the gut wall [167]. EMT of intestinal epithelial cells yields a population of mesenchymal cells that activate resident fibroblasts and differentiate into myofibroblasts, promoting fibrotic strictures in Crohn's disease and UC. In the airways, repeated epithelial injury in chronic asthma or CRS similarly leads to EMT‐driven remodeling. The airway epithelium in asthma, usually a tight barrier, becomes disrupted; some epithelial cells transition into fibroblast‐like cells that migrate beneath the basement membrane. This contributes to subepithelial fibrosis and smooth muscle hyperplasia, thickening the airway wall [168]. A good example of this is *lamina reticularis* thickening beneath the basal lamina in asthmatic bronchi, which is readily observed at the time of first diagnosis as shown in biopsies taken on the day of initial diagnosis of asthma in children [5]. The result is airway narrowing and loss of elasticity, manifesting as irreversible airflow limitation in severe asthma. In allergic diseases, EMT is increasingly recognized as a feature of chronic tissue changes. Atopic dermatitis (chronic eczema) skin shows areas of EMT indicated by keratinocytes expressing mesenchymal markers, which correlates with barrier breakdown and lichenified (thickened) skin lesions [169]. Eosinophilic esophagitis (EoE), an allergic inflammation of the esophagus, provides a striking example: IL‐13–driven inflammation in EoE causes esophageal epithelial cells to downregulate E‐cadherin and acquire mesenchymal markers, contributing to tissue stiffening and dysmotility. Notably, a clinical trial in EoE found that an anti–IL–13 antibody could restore epithelial E‐cadherin and reduce vimentin‐positive cells, effectively reversing EMT biomarkers in the esophageal lining [172]. This demonstrates that EMT is not only present in these diseases but is functionally important in barrier dysfunction and can be therapeutically targeted. Across these conditions—from IBD in the gut to asthma/CRS in the airways to AD and EoE—EMT under chronic inflammatory conditions leads to compromised barriers, infiltration of immune cells, and progressive fibrosis. We presume that this second layer of fibrotic barrier develops in epithelial barrier leaky individuals to protect the deeper tissues from environmental pollutants, the microbiome, allergens, and toxins, which become pathologic in the presence of chronic damage to the epithelial barriers. These changes often perpetuate inflammation and can cause organ dysfunction (such as strictures, bronchial hyperresponsiveness, or impaired peristalsis), thereby driving the chronic nature of the disease.

## Phenotypes and mechanisms of epithelial barrier leakage

More than 2000 years ago, Hippocrates said, ‘What diseases the medicines cannot heal, iron heals; what iron cannot heal, fire heals; and what fire cannot heal, these must be considered incurable’, which means if the medicine is not sufficient, particularly for surgical wounds and abscesses, surgical drainage, full cleaning of the wound with further drainage by heated oils will be needed as a further step after surgical drainage. What he suggested was that surgical drainage takes place in the epithelium. If there is epithelial damage and a breach in the barrier with ongoing subepithelial inflammation, the response of the tissue is to *expel, drain*, or *wash away* the insulting substance, inflammatory cells, and cytokines to decrease the inflammatory burden [5, 13].

Epithelial cells, including enterocytes, actively ferry luminal macromolecules across the barrier via vesicular transcytosis (Fig. 3C), a crucial transport route that is both TJ‐independent and size‐flexible. In response to epithelial damage and subsequent subepithelial inflammation, an adaptive tissue mechanism involving increased epithelial permeability may be triggered to facilitate the clearance or ‘washing away’ of noxious substances and inflammatory mediators into the lumen, potentially reducing the local inflammatory burden [5]. Studies investigating type 2 inflammation, relevant to conditions like asthma, have demonstrated that type 2 innate lymphoid cells (ILC2s), primarily through the action of interleukin‐13 (IL‐13), can directly induce bronchial barrier disruption by downregulating crucial TJ proteins, such as Occludin and ZO‐1 [13]. This leads to functionally compromised barrier integrity, evidenced by reduced TEER, increased paracellular flux, and the *in vivo* leakage of tissue proteins like α2‐macroglobulin and transferrin into the airway lumen [13]. Although this IL‐13‐driven leakiness is often implicated in asthma pathogenesis by potentially increasing allergen penetration and contributing to chronicity, the resulting outward flux of endogenous molecules physically aligns with the conceptual possibility of draining inflammatory components from the tissue across the compromised epithelial barrier [5, 13].

### The pore pathway

The pore pathway refers to paracellular leaks of small ions and solutes through size‐ and charge‐selective channels in TJs (Fig. 3D). These channels are formed by claudin proteins; for example, claudin‐2 forms cation‐selective pores that increase conductance for small molecules. In inflammatory conditions, pore‐forming claudins can be upregulated, enhancing this pathway. A striking example is in UC, in which the Th2 cytokine IL‐13 is overproduced and drives a ~ 10‐fold increase in claudin‐2 expression, significantly reducing TEER [173]. This IL‐13‐induced pore‐type leak allows excess Na^+^ and water efflux into the gut lumen, contributing to diarrhea. Although this contributes to chronic leakiness, in principle, it functions to drain the inflammation in the deeper colon tissues and decreases the inflammatory burden [5]. Parallel changes are seen in UC patient biopsies (claudin‐2 up nearly 956% over normal) [173], underscoring the pore pathway's role in disease. Other epithelial barriers have analogous mechanisms—for instance, claudin shifts in airway or skin epithelium can alter ion permeability—but the pore pathway is most clearly implicated in intestinal disorders like UC. Importantly, pore‐size leaks predominantly allow small nutrient molecules or electrolytes to cross, so while they disturb fluid balance, larger antigenic molecules and microbes remain mostly restricted. Thus, pore pathway dysfunction often leads to symptoms like fluid loss (as in UC) without immediately provoking massive immune activation.

### The leak pathway

The leak pathway involves a nonspecific loosening of TJs that permits larger molecules (up to tens of kDa) to permeate between cells. This can occur via cytokine‐driven junctional changes or direct injury to junction proteins. Pro‐inflammatory cytokines are a common cause: for example, TNF‐α and IFN‐γ synergistically disrupt intestinal TJs by activating myosin light‐chain kinase (MLCK), which pulls the peri‐junctional actin ring and opens the junctional seal [174]. *In vitro*, TNF on IFN‐γ–primed intestinal monolayers caused a steep drop in TEER and ‘unzipping’ of TJ strands—effects that were prevented by MLCK inhibition [174]. Notably, this form of barrier dysfunction does not require cell death; junctional proteins (occludin, ZO‐1, etc.) are redistributed or internalized, creating large pores between still‐viable cells [174]. Such cytokine‐induced leakiness is prominent in Crohn's disease, where elevated TNF levels lead to high paracellular permeability and allow antigens/bacterial products to cross the gut lining, correlating with systemic immune activation [174]. Environmental factors can also trigger the leak pathway. A classic example is the house dust mite allergen Der p1 in airways, a protease that cleaves epithelial TJ proteins. Der p1 rapidly cuts occludin (and claudin‐1) in bronchial epithelium, causing TJ breakdown and a surge in macromolecular permeability [175]. This enables allergens to traverse the airway barrier and access submucosal immune cells, facilitating allergic sensitization in asthma. Likewise, in AD skin, the Th2 cytokines IL‐4 and IL‐13 downregulate key TJ components, such as claudin‐1, weakening the junctional seal [176]. Skin models exposed to IL‐4/IL‐13 show reduced claudin‐1 and consequent barrier impairment, allowing ingress of environmental antigens and irritants. These leak‐pathway defects in AD are linked to flare‐ups, as more allergens and microbes penetrate the loose junctions to activate cutaneous immune responses. In summary, the leak pathway—detectable by increased flux of larger tracers (e.g., 4 kDa dextran)—is a common consequence of inflammation and toxins. It underlies conditions from IBD (where it can presage relapses) to asthma and eczema, by permitting abnormal antigen passage and immune triggering.

### Tricellular junction disruption (macromolecule leak at three‐cell contacts)

At points where three epithelial cells meet (tricellular junctions), a specialized structure seals the tiny central gap. Tricellulin is a critical protein concentrating at tricellular contacts to prevent leakage. If tricellulin or associated proteins (e.g., angulin) are compromised, a distinct leak pathway opens preferentially for large solutes. In cell experiments, lowering tricellulin levels dramatically increases paracellular permeability to macromolecules while only mildly affecting small ion flow [177]. This is because the ‘central tube’ at tricellular contacts—normally too narrow to contribute to ion conductance—can widen enough to pass molecules > 4 kDa when not sealed by tricellulin [177]. In contrast, abundant tricellulin closes this route. Such a mechanism has been implicated in diseases like UC. Active UC mucosa exhibits significantly reduced tricellulin expression, which is reversible upon remission. A 2018 study found that IL‐13 signaling (via IL‐13Rα2) downregulates tricellulin in the intestinal epithelium, increasing the flux of macromolecular tracers across the barrier [178]. In UC patients, this tricellular leak may allow large antigens or bacterial products to permeate the gut lining, fueling mucosal immune responses. Interestingly, Crohn's disease (a Th1/Th17‐dominant IBD) did not show tricellulin loss in that study, highlighting that tricellular defects might be more relevant to Th2‐associated inflammation (as seen in UC's atypical Th2 profile). Although less studied in skin and airway, tricellular TJs exist in those tissues and could be targeted by similar mechanisms—for example, IL‐13 in allergic asthma or AD might hypothetically loosen tricellular contacts as well. Overall, tricellular junction disruption creates a pathway for large molecules (e.g., dietary antigens and pathogens' products) that are normally too big to cross. When this occurs in the gut or other organs, it can lead to heightened immune surveillance and inflammation due to the abnormal influx of luminal macromolecules.

### Apoptotic or mechanical cell extrusion

Epithelial barriers undergo continuous cell turnover—old or damaged cells are extruded and replaced by neighbors. Apoptotic or mechanical cell extrusion can transiently disrupt the barrier if the expelling cell leaves behind a gap. In healthy epithelia, an intricate remodeling of TJs accompanies extrusion to preserve barrier integrity. Live imaging in mice has shown that when tumor necrosis factor induces extensive intestinal epithelial shedding, surviving neighbors mobilize their TJ proteins to form a ‘funnel’‐shaped structure beneath the extruding cell [179]. ZO‐1 and occludin relocate along lateral membranes, and together with actomyosin contraction, they seal the opening as the apoptotic cell exits [179]. Thanks to this dynamic TJ expansion, the gut can withstand a certain rate of cell loss without gross leakage. However, even in these adaptive events, there are momentary leaks—for instance, local conductance mapping reveals a spike in ion permeability directly under shedding cells [179]. If cell extrusion is excessive or dysregulated, the compensatory mechanisms fail. In inflammatory conditions like graft‐versus‐host disease or severe Crohn's flares, epithelial apoptosis is greatly increased, leading to multiple synchronous extrusions. Under such strain, small gaps (‘microerosions’) can remain unsealed, forming localized ‘unrestricted pathways’ for paracellular passage that permit paracellular leakage of solutes and even microbes (Fig. 3D). Experimentally, high‐dose TNF in mice caused rapid epithelial shedding that, despite active junctional remodeling, resulted in measurable drops in barrier function [179]. Likewise, mechanical stresses can induce extrusion‐based leaks: in asthma, for example, chronic coughing or allergen exposure can damage airway epithelial cells, causing them to slough off. The shed cells (seen as Creola bodies in sputum) reflect epithelial gaps that can let inhaled particles or pathogens penetrate more deeply. In summary, accelerated cell extrusion due to apoptosis or mechanical injury produces transient leak phenotypes. These are often patchy and short‐lived, but can cumulatively compromise the barrier if cell loss outpaces repair, thereby exposing underlying tissues to antigens.

### Erosive barrier loss (full‐thickness breaches)

The most severe phenotype is erosive barrier loss, where regions of epithelium are denuded entirely, leaving the basement membrane or submucosa directly exposed. In this scenario, there is essentially no cellular barrier—a ‘leak’ in the form of an outright wound or ulcer. All epithelial organs can suffer erosions under extreme conditions. In the gut, erosive lesions are hallmark features of severe IBD. For instance, during a fulminant UC flare and in severe cases of Crohn's disease, widespread epithelial necrosis leads to ulceration of the colonic mucosa. These ulcerated areas permit unrestrained exchange of material: luminal bacteria and antigens flood into tissue, while fluids and proteins leak out. The result is often acute immune activation with intense inflammation (and systemically, fever and elevated C‐reactive protein). Even in Crohn's disease, microscopic erosions can precede visible ulcers—one study noted that increased intestinal permeability can precede clinical disease and predicts relapse [174]. Erosive loss of barrier is also seen in EoE and chronic reflux esophagitis, where chronic inflammation plus mechanical stress (e.g., swallowing food boluses against a stiff and inflamed esophagus) can strip away patches of the stratified epithelium. EoE patients often exhibit dilated intercellular spaces and weakened epithelial adhesion due to IL–13–mediated loss of structural proteins (filaggrin and desmoglein‐1). In a 2014 study, knockdown of DSG1 (desmoglein‐1) in esophageal epithelial cultures caused cells to separate and barrier function to drop, even when viability was maintained [180]. This suggests that in EoE, chronic Th2 inflammation can disrupt cohesion so that sheets of epithelium detach (erosion), manifesting endoscopically as friable ‘crepe‐paper’ mucosa. Skin barrier erosions are common in severe AD: repeated scratching and Staphylococcus infection can create open sores where the epidermis is breached. Bullous skin diseases, characterized by sloughing of the epidermal layer, are prototypical examples of erosive barrier loss. Pemphigus vulgaris is an autoimmune disease in which autoantibodies against desmoglein lead to disruption of desmosomes, formation of vesicles, and erosion of the epithelium [181]. These sites allow allergens and microbes to penetrate unabated, often leading to localized infection or even systemic effects (e.g., eczema herpeticum when viral entry occurs through broken skin). Notably, an erosive barrier loss creates an entry portal for organisms—bacterial translocation from gut to blood can occur through intestinal ulcers, and in chronic liver disease patients, leaky gut ulcers are a known source of sepsis. Clinically, sealing erosions (with medications or physical barriers) is crucial, as even small ulcers can perpetuate inflammation by providing a route for continuous antigen influx. Thus, erosive barrier defects represent an extreme leak pattern underlying the most acute and dangerous phases of barrier‐related diseases, from IBD with profuse bleeding to refractory EoE and infected eczematous dermatitis. In all cases, restoring epithelial coverage is necessary to re‐establish homeostasis.

## Peri‐epithelial inflammatory pathways

### Innate immune response

Epithelial barrier integrity is not only threatened by external toxic substances but is also compromised by underlying subepithelial inflammation [182]. Activated epithelial cells release cytokines, including alarmins like IL‐25, IL‐33, and TSLP, which are potent drivers and amplifiers of allergic inflammation [182]. Open TJs, while potentially serving to drain inflammatory mediators from below, simultaneously permit easier access of foreign substances into deeper tissue layers [182]. Both type 1 and type 2 inflammatory responses impact epithelial barriers. Type 1 inflammation can directly injure epithelial cells through apoptosis induction, while type 2 inflammation, mainly driven by T‐helper 2 (Th2) and innate lymphoid type 2 (ILC2) cells releasing IL‐4 and IL‐13, is known to disrupt bronchial, sinus, and skin epithelial barriers [8, 12, 13]. It is crucial to consider that exposure to different barrier‐disrupting substances might exert synergistic, rather than merely additive, effects due to shared toxicity mechanisms. Moreover, individuals with pre‐existing epithelial inflammation may be susceptible to barrier damage at lower concentrations of these substances.

A proposed cascade of events linked to the epithelial barrier hypothesis begins with barrier damage, often induced by environmental exposures [1]. This initial insult can lead to microbial dysbiosis, favoring opportunistic pathogens over commensals and facilitating the transepithelial translocation of these microbes and potentially commensals into the subepithelium [1, 183]. This translocation stimulates immune responses, leading to subepithelial or submucosal inflammation [1]. Persistent inflammation and immune responses against both pathogens and potential commensals can further drive dysbiosis and decrease microbial biodiversity [1, 184]. For instance, the association between *Staphylococcus aureus* colonization and anti‐*S. aureus* IgE with the severity of asthma, AD, and CRS appears more prominent now than decades ago [185, 186, 187, 188, 189]. This chronic inflammatory state promotes dysregulated tissue repair, remodeling (such as epithelial basement membrane thickening, potentially as a secondary barrier attempt), and fibrosis [1]. Systemic consequences may arise from the migration of inflammatory cells from leaky barrier sites or through sustained low‐level systemic inflammation and immune activation, potentially contributing to the development or exacerbation of various chronic diseases [1].

Acute epithelial injury launches a peri‐epithelial ‘Type 0’ circuit initiated by DAMPs. Necrotic or stressed epithelial cells release nuclear alarmins like IL‐33 and IL‐1α, as well as ATP and HMGB1, which activate sentinel immune cells (resident macrophages, dendritic cells, and mast cells) via pattern‐recognition receptors [190, 191]. These innate sensors assemble inflammasomes that activate caspase‐1, leading to pyroptotic cell death and release of IL‐1β and IL‐18 [191, 192]. The result is an early burst of IL‐1 family cytokines that recruit neutrophils and amplify inflammation. IL‐1β itself increases local endothelial and epithelial permeability, and epithelial pyroptosis creates physical gaps in the barrier [191]. Meanwhile, epithelial‐derived IL‐33 and TSLP directly alert group 2 innate lymphoid cells (ILC2s), mast cells, and basophils, skewing local immunity toward Type 2 responses [190]. This Type 0 circuit compromises barrier integrity through rapid TJ disruption and cell loss—a priming event that sets the stage for downstream adaptive immunity. Notably, in diseases like AD, mechanical epithelial damage (scratching) leads to repetitive DAMP release (IL‐33 and TSLP), fueling an innate cycle of barrier destruction and inflammation [190].

### Type 1 inflammation and epithelial barriers

Following an initial DAMP‐driven phase, an immune response can polarize toward a Type 1 circuit if antigen‐presenting cells provide IL‐12 and IFN‐α/β signals. Type 1 peri‐epithelial inflammation is classically triggered by intracellular pathogens (viruses and certain bacteria) and characterized by IFN‐γ and TNF‐α production from Th1 cells, ILC1s, and NK cells. IFN‐γ‐activated dendritic cells and macrophages amplify this circuit by secreting IL‐12 and IL‐18, creating a positive feedback that sustains IFN‐γ release [193]. These Type 1 cytokines act on epithelial barriers to potentiate leakiness: IFN‐γ signaling in epithelial cells induces endocytosis of TJ proteins (occludin, claudin, and JAM‐A), leading to increased paracellular permeability [194]. In parallel, TNF‐α released by activated macrophages and Th1 cells triggers epithelial cell apoptosis and initiates a ‘leak’ pathway in TJs [139]. Indeed, TNF‐α not only induces programmed cell death in barrier epithelial cells, but also opens paracellular junctional channels via cytoskeletal contraction [139]. The combined assault of IFN‐γ and TNF causes focal epithelial loss and loosened TJs, as seen in Crohn's disease, in which Th1 cytokines drive mucosal ulceration. Cell death mechanisms like apoptosis and pyroptosis are prominent: IFN‐γ and TNF synergistically activate caspases and inflammasomes in the epithelium, causing shed cells and exposing the lamina propria [139, 195]. A downstream loop is established as a barrier breach that allows microbial products to penetrate, stimulating more IL‐1β and IL‐12 release from sentinel cells and perpetuating chronic Type 1 inflammation. For example, in IBD of the Crohn's type, granulomas rich in IFN‐γ and IL‐12 form alongside deep epithelial fissures, and blockade of TNF‐α can rescue TJ integrity [139]. Thus, Type 1 circuits compromise barriers via cytokine‐driven junctional disassembly and cell death, linking infections and autoimmunity to epithelial dysfunction.

### Type 2 inflammation and epithelial barriers

In contrast, Type 2 peri‐epithelial circuits are launched by epithelial encounters with allergens, parasites, and environmental irritants. Damaged or exposed barrier cells release alarmins (IL‐33, IL‐25, and TSLP) that activate local dendritic cells, mast cells, and ILC2s, skewing the response toward a Th2 phenotype [190]. These sentinel cells produce IL‐4, IL‐5, IL‐13, and other Type 2 cytokines, which orchestrate eosinophilic inflammation and mucus hypersecretion. Crucially, IL‐4 and IL‐13 from Th2 cells and ILC2s directly impair epithelial TJs and differentiation. IL‐4/IL‐13 signaling in keratinocytes and mucosal epithelium downregulates key barrier proteins like filaggrin, loricrin, claudins, and E‐cadherin [190]. In the airway and sinus epithelium, IL‐4/IL‐13 reduce transepithelial resistance and junctional adhesion molecules, effectively opening paracellular ‘pore‐pathways’ that increase permeability [139, 190]. Likewise in skin, IL‐4/IL‐13 suppress filaggrin and lipid production, raising pH and permitting *Staphylococcus aureus* colonization [190]. Recruited eosinophils further damage the barrier by releasing cytotoxic granule proteins (major basic protein and eosinophil peroxidase) and proteases. These eosinophil mediators directly disrupt TJ function and stratum corneum integrity, creating microscopic breaches [190]. The result is a self‐amplifying loop: barrier defects allow deeper allergen and microbe penetration, which sustains epithelial alarmin release and chronic Type 2 inflammation [139, 190]. This circuit underlies diseases like asthma, AD, and EoE. In asthma, epithelial exposure to allergens (dust mite proteases and pollen) induces IL‐33/TSLP, driving ILC2‐mediated IL‐13 production that increases airway epithelial permeability and goblet cell metaplasia [190]. In AD, an IL‐4/IL‐13‐rich milieu causes epidermal junctional weakening and loss of the moisture barrier, while IL‐31 from Th2 cells provokes itching that leads to further barrier trauma (the itch‐scratch cycle) [190]. In EoE, Th2 cytokines (notably IL‐13) induce an esophageal epitheliopathy: IL‐13 downregulates desmoglein‐1 in the esophageal epithelium, loosening cell–cell adhesion and contributing to epithelial barrier leak [196]. Thus, Type 2 circuits center on a cytokine‐driven barrier defect—via junctional dilatation, impaired differentiation, and cell loss—that is characteristic of allergic diseases.

### Type 17 inflammation and epithelial barriers

A third peri‐epithelial circuit, Type 17, emerges in response to extracellular bacteria and fungi breaching barrier defenses. This circuit is mediated by IL‐17‐producing cells (Th17 CD4^+^ T cells, γδ T cells, and ILC3s) activated under the influence of IL‐23, IL‐1β and IL‐6 from antigen‐presenting cells [193]. Signature cytokines IL‐17A, IL‐17F, IL‐22, and GM‐CSF define the Type 17 milieu [193]. IL‐17 profoundly reshapes the epithelial microenvironment: it induces chemokines (CXCL1 and CXCL8/IL‐8) and G‐CSF from epithelial and stromal cells, driving massive neutrophil recruitment to the barrier [193]. Neutrophils in turn release ROS and proteases (elastase, cathepsin G, and MMPs) that cause collateral tissue damage—cleaving extracellular matrix and junctional proteins and even activating more inflammatory cytokines (pro‐TNF and pro‐IL‐36) in the tissue [191]. Indeed, neutrophil‐derived IL‐1β and IL‐36α act on local cells to amplify Th17 responses, creating a feed‐forward loop [191]. Barrier integrity is compromised as IL‐17 and associated cytokines alter epithelial biology: IL‐17 and IL‐1β together downregulate epithelial adhesion molecules and TJ components, leading to junctional disorganization [191]. For example, in psoriatic skin, IL‐17 and IL‐1 signals together reduce the expression of claudins and E‐cadherin, and TJ dysregulation is detectable even in early lesions [191]. IL‐22 from Th17/Th22 cells further contributes by inducing keratinocyte hyperproliferation while inhibiting their differentiation, resulting in a thick but functionally leaky epithelium [197]. At mucosal sites, IL‐17 can paradoxically strengthen acute defenses (promoting mucus and antimicrobial peptides), but in chronic Type 17 inflammation, these protective effects are offset by excessive neutrophil‐driven damage and impaired barrier repair [193]. In the intestine, for instance, persistent IL‐23/Th17 activation disrupts the epithelial TJ seal and goblet cell function, allowing microbial translocation that perpetuates colitis [193]. This is evident in Crohn's disease and some cases of UC, where IL‐17/IL‐22 and TNF form an inflammatory axis causing deep crypt damage and erosions. In psoriasis, a prototypical Type 17 disease, IL‐17 and IL‐22 lead to epidermal hyperplasia and neutrophil microabscess formation, with measurable loss of barrier function (increased transepidermal water loss and penetrance of antigens) [191]. Thus, the Type 17 circuit compromises epithelial barriers through cytokine‐mediated junctional disruption and neutrophil‐induced tissue injury, tying mucocutaneous infection control to autoimmune pathology.

Notably, these peri‐epithelial inflammatory circuits interconnect, and chronic barrier diseases often involve multiple overlapping types [1, 139]. An initial Type 0 DAMP response can bias the immune milieu toward Type 1, 2, or 17 depending on context—for example, epithelial IL‐33 release (Type 0) favors a Type 2 cascade, whereas IL‐1β release favors neutrophils and Th17 differentiation [190, 191]. Once established, each circuit creates a pro‐inflammatory microenvironment that can recruit elements of the others. In AD, a primarily Type 2 disorder, barrier disruption and microbial dysbiosis (e.g., *S. aureus* colonization) can provide PAMP signals that induce local IL‐1β and IL‐17, layering a Type 17 component onto chronic eczema [190, 191]. Conversely, in psoriasis or Crohn's disease (Type 17/Type 1 conditions), periods of tissue damage can release IL‐33 and TSLP, acutely activating Type 2‐like wound‐healing responses that coexist with the Th17/Tc17 milieu. Thus, real‐world inflammatory lesions are a mosaic: Type 1 and Type 2 circuits often coactivate (both IFN‐γ and IL‐13 are elevated in inflamed atopic skin and gut) [139, 173], and Type 17 may emerge when Type 1 or 2 responses fail to clear pathogens (as in steroid‐resistant neutrophilic asthma). These interactions create vicious cycles in the peri‐epithelial niche. Barrier breach leads to microbe ingress and antigen exposure, which triggers further immune activation and cytokine release, in turn causing more barrier damage [139]. A persistent loop of ‘injury–inflammation–injury’ develops, with each circuit's mediators aggravating the others. For instance, IL‐4/IL‐13 can recruit neutrophils via IL‐8 from epithelial cells, IL‐17 can stimulate macrophages to produce IL‐1β and TNF, and IFN‐γ can induce epithelial cells to secrete the alarmin IL‐33 under stress. Without counter‐regulation, these feedback loops lock the system in a state of chronic inflammation and defective barrier repair [139]. In summary, while Type 0, 1, 2, and 17 responses are described separately, in chronic disease, they form an integrated network of cytokine loops that continually erode epithelial integrity.

### Acute and chronic epithelitis in the initiation and maintenance of chronic peri‐epithelial inflammation

The concept of ‘chronic epithelitis’, characterized by sustained pro‐inflammatory cytokine release and barrier damage, emerges from exposure to compounds like food emulsifiers and detergents. Environmental toxic insults to epithelium can induce significant expression of alarmins, IL‐33 and TSLP, together with inflammasome activation, expression of pro‐inflammatory cytokines and multiple chemokines, and eosinophilic inflammation in tissues like the airways, gut, and esophagus, and skin [16, 20, 21, 57, 86, 198, 199]. Mouse studies demonstrated that even brief intranasal exposure to common detergents or surfactants induced eosinophilic airway inflammation involving IL‐33 and ILC2 activation, an effect mitigated in mice lacking adaptive lymphocytes and ILCs or IL‐33 [86]. Similarly, low‐dose SDS exposure in models relevant to EoE decreased esophageal barrier integrity, increased IL‐33, and promoted esophageal eosinophilia and inflammation [57], suggesting detergents in products like toothpaste could be triggers.

## Genetic and epigenetic changes linked to epithelial barriers

Individual susceptibility is significantly modulated by genetic factors. Mutations in genes encoding crucial epithelial barrier proteins are linked to asthma, AD, and food allergies [200, 201, 202, 203]. For instance, rare variants impairing desmosome structures (desmoplakin and periplakin) contribute to EoE [204], mutations in AP1S1 cause congenital diarrhea via intestinal barrier defects [205], and mutations in CLDN10B affect sweat gland barriers [206]. Congenital tufting enteropathy, typically resulting from mutations in the EPCAM gene encoding an epithelial cell adhesion molecule, involves disrupted enterocyte adhesion and differentiation, leading to pathognomonic ‘tufts’, a profoundly compromised intestinal barrier and severe, intractable lifelong diarrhea [207]. Polymorphisms in genes for alarmins (TSLP and IL‐33) and type 2 cytokines (IL‐4 and IL‐13) influence asthma risk and severity [202], although many genetic contributors remain unidentified [208]. Filaggrin deficiency, known in AD, may also promote Th2 inflammation in airways by affecting E‐cadherin expression and enhancing IL‐33/TSLP release, potentially contributing to asthma [209].

Epigenetic modifications add another layer of regulation. Studies show altered histone acetylation/deacetylation patterns in asthmatic bronchial epithelial cells, with increased levels of HDACs 1 and 9, and SIRTs 6 and 7 [12, 210]. IL‐4 and IL‐13 can drive these changes, and inhibiting HDACs can improve barrier integrity in asthmatic cells by restoring TJ molecule synthesis [12]. Altered DNA methylation patterns affecting pro‐inflammatory and regulatory cytokine gene expression have also been observed [210]. These epigenetic changes can impact the healing capacity of epithelial stem cells and contribute to the chronicity of barrier defects and associated inflammation [12, 210]. Epigenetic and metabolic reprogramming in epithelial and immune cells locks in disease‐driving pathways—evident in enduring psoriasis and IBD lesions, where an entrenched inflammatory ‘memory’ resists healing [211, 212].

## Role of the microbiome

Microbial dysbiosis is a central element of the epithelial barrier theory. Barrier damage facilitates colonization by opportunistic pathogens like *S. aureus* in AD [213], or facultative pathogens like *Streptococcus pneumoniae*, *Haemophilus influenzae*, and *Moraxella catarrhalis* in respiratory conditions [15, 214, 215], loss of beneficial commensals, and subsequent bacterial translocation [1, 56]. This translocation is implicated in driving autoimmune and chronic inflammatory conditions [216, 217, 218, 219, 220, 221, 222, 223, 224]. The altered host‐microbiota interactions lead to abnormal mucosal immune responses, potentially skewing toward Th17, Th1, or Th2 responses, downregulating regulatory T cells, and dysregulating humoral immunity [184, 225]. Persistent exposure to harmful factors might also impair epithelial cell metabolism and regenerative capacity [226].

The immune system's interaction with microbes is complex. Normally, host defenses prevent opportunistic infections. However, in chronic inflammatory settings, bacteria can manipulate immune responses, sometimes evading detection (e.g., by inactivating Toll‐like receptors) [227, 228]. *S. aureus*, for example, employs numerous strategies to evade neutrophil defenses, including inhibiting chemotaxis, resisting phagocytosis, killing neutrophils, and neutralizing antimicrobial peptides and proteases using factors like Eap proteins [229, 230].

Diet significantly influences the interplay between the epithelium and the immune system and the microbiome. Fiber deprivation in mice led to an increase in *Akkermansia muciniphila*, which, in the absence of fiber, exacerbated food allergy symptoms, possibly through anti‐commensal IgE and innate type 2 responses [231]. Commensal protozoa like *Trichomonas musculis* can stimulate tuft cells via succinate to release IL‐25, activating ILC2s to produce IL‐13, thereby altering Paneth cell function and microbial composition [232]. Specific microbial strains can also drive pathology; high virulence *Candida albicans* strains that dominate the gut in IBD patients aggravate inflammation via IL‐1β, potentially involving toxins like candidalysin.

Barrier dysfunction directly impacts microbial niches. In AD‐like skin, structural and metabolic changes facilitate the overgrowth of the commensal fungus *Malassezia* [231, 233]. There are also systemic consequences mediated through gut‐axis communication. Intestinal Candida dysbiosis in mice enhanced Th2 responses to airway allergens (HDM), associated with increased lung ILC2s, demonstrating a gut–lung connection [234]. Conversely, skin damage can alter gut microbial composition and function, potentially exacerbating intestinal inflammation via a skin–gut axis [235]. Pathogens can also actively remodel their niche; *Pseudomonas aeruginosa* uses its toxin LasB to induce epithelial amphiregulin and type 2 inflammation, promoting mucin production, which serves as its energy source, thereby facilitating colonization and allergic sensitization [236].

Loss of microbial diversity is a common feature of dysbiosis linked to Western lifestyles and numerous diseases, including dermatological conditions [237, 238, 239]. Host factors are crucial for maintaining diversity; for example, neutrophil recruitment via CXCR2 is essential for controlling the periodontal microbiome and preventing inflammatory bone loss [240]. Similarly, epithelial TLR4 signaling helps maintain gut homeostasis during pancreatitis, possibly involving interactions with beneficial bacteria like *Lactobacillus reuteri* [241]. Therapeutic modulation is also possible; a protease inhibitor from *Trichinella spiralis* (rTsSPI) showed potential to reduce gut inflammation, shift macrophage polarization, increase IL‐10, and enhance gut microbial diversity in mice [242].

Persistent epithelial barrier defects and the resulting chronic inflammation drive tissue‐specific pathology [1]. In EoE, overexpression of IL‐33 alone can induce EoE‐like inflammation and remodeling, likely via the IL‐13 pathway [243]. As mentioned, common household detergents and dishwashing agents disrupt airway, skin, and esophageal barriers, inducing inflammatory responses and cell death pathways [16, 20, 22, 57]. Air pollutants contribute by activating type 2 pathways, promoting epithelial cytokine release (IL‐33 and TSLP), oxidative stress, and pro‐inflammatory mediators (IL‐6, IL‐8, and IL‐1β) relevant to asthma [244]. In CRS with nasal polyps, type 2 cytokines orchestrate inflammation, immune cell recruitment, tissue remodeling, and chronic barrier dysfunction [245]. Aeroallergen exposure can trigger ATP/ADP release, activating purinergic receptors (like P2Y1) on epithelial cells to induce IL‐33 and HMGB1 release, contributing to asthma pathogenesis [246]. This highlights a recurring theme: barrier disruption, often environmentally triggered, leads to alarmin/cytokine release, driving inflammation and disease‐specific pathology. The ongoing interplay between barrier leakiness and peri‐epithelial inflammation creates a vicious cycle that perpetuates tissue damage and dysfunction [1].

## Measuring epithelial barrier status

**Fig. 4:**
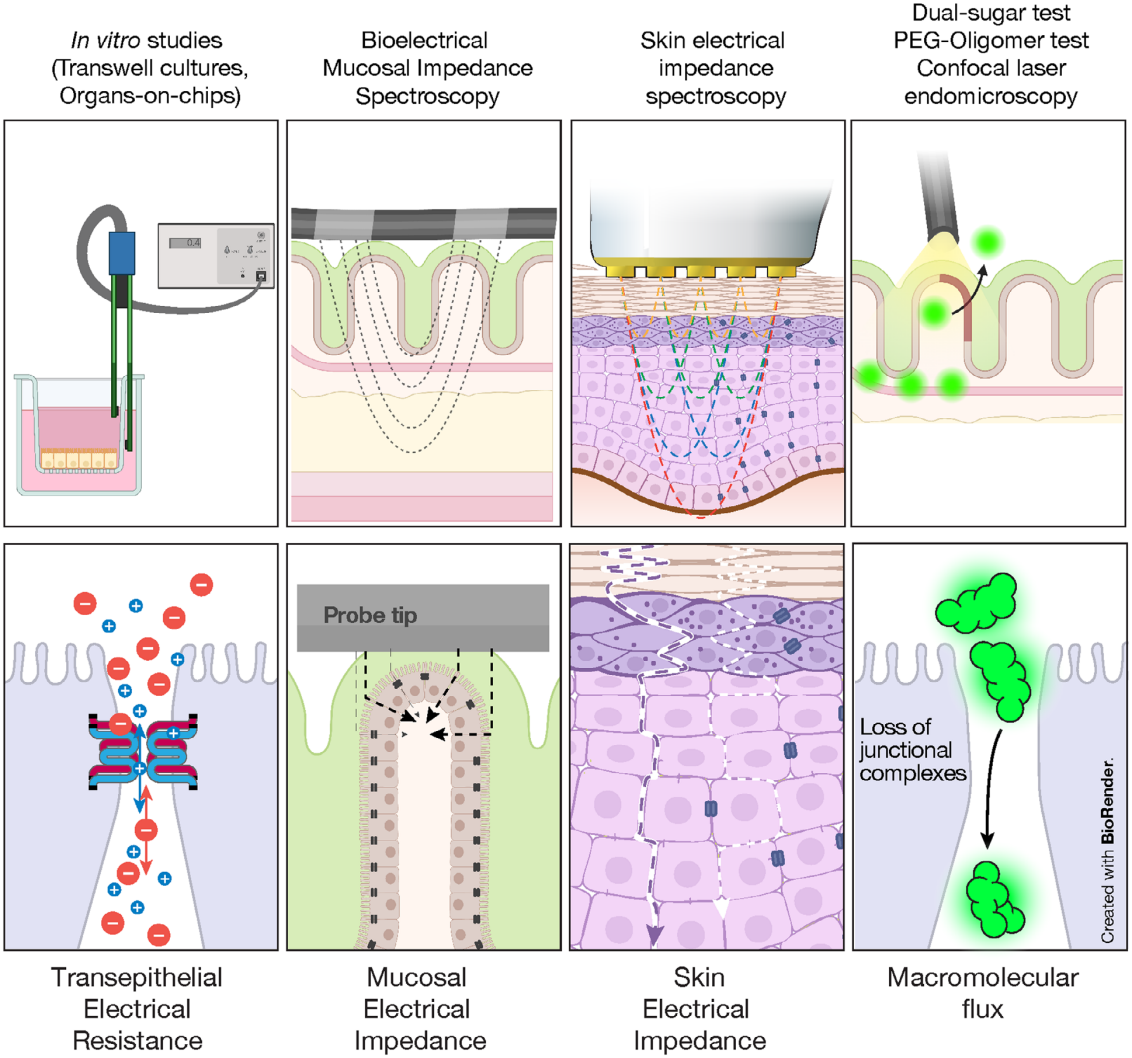
Representative assays for quantifying epithelial barrier integrity and their underlying mechanisms: transepithelial electrical resistance (TEER) measurements in Transwell cultures or organs‐on‐chips determine ionic conductance across tight junctions by applying a low‐amplitude alternating current; mucosal and skin bioelectrical impedance spectroscopy injects frequency‐swept currents through the epithelium and subepithelium to record complex impedance changes; confocal laser endomicroscopy, together with dual‐sugar or polyethylene glycol (PEG) oligomer permeability tests, monitors paracellular passage of fluorescent or labeled macromolecules to detect junctional disruption.

Accurate evaluation of epithelial barrier function now spans quantitative biophysics, functional permeability testing, and molecular readouts (Table 4, Fig. 4). *In vitro* and *ex vivo*, TEER remains the reference standard: a high‐throughput, nondestructive readout of TJ integrity that is easily integrated into organ‐on‐chip platforms. The usage of primary cells obtained from tissues of affected individuals and induced pluripotent stem cells has brought a new perspective to the area. A sustained fall in TEER is usually paralleled by increased paracellular tracer flux, and both parameters tightly correlate with morphological junctional disruption across gut, airway, renal, and blood–brain barrier models [247]. In patients, the functional leak is best captured by differential sugar absorption, the lactulose–mannitol ratio. Meta‐analysis of over 30 studies confirmed that this simple 5‐h urine test discriminates healthy subjects from active coeliac or Crohn's disease with large effect sizes, supporting its value as a bedside surrogate of intestinal ‘leakiness’ [248].

**Table 4:** Assays for quantifying epithelial barrier integrity. BAL, bronchoalveolar lavage; CLE, confocal laser endomicroscopy; EIS, electrical impedance spectroscopy; OoC, organonchip; TJ, tight junction.

Modality	Principle	Prep/scale	Readout (units)	Applications^a^	References
TEER	Ion conductance across TJs	Monolayers, OoCs	Ω·cm^2^ (real time)	S, T	[247]
Tracer flux (FITCdextran, ^51^CrEDTA)	Fickian diffusion through paracellular pores	Monolayers, excised mucosa	μg·cm^−2^·h^−1^	S, Q	
Sugar test (Lactulose/Mannitol)	Differential trans vs. paracellular uptake	Human intestine *in vivo*	L : M ratio	D, M	[248]
CLE	Real‐time fluorescein leakage imaging	GI mucosa	Pixel leak index	D	[249]
EIS	Complex impedance of stratum corneum or mucosa	Skin probe, esophageal catheter	(kΩ)	D, T	[250]
Baseline mucosal impedance	Baseline ion permeability	Esophageal lumen	Ω profile	D	[253]
Biomarkers	Shedding or alarmin release	Serum, stool, BAL	ng·mL^−1^	P, E	[1, 22]

^a^ Key codes—S: safety/toxicology screening; T: tight‐junction gene manipulation studies; Q: quantitative permeability kinetics; D: diagnostic use in humans; M: therapy monitoring; P: population biomarker; E: endpoint in interventional trials.

Imaging and biomarker technologies add spatial and mechanistic resolution. Confocal laser endomicroscopy (CLE) allows real‐time, *in vivo* visualization of fluorescein leakage through epithelial gaps; in a prospective study, it identified barrier dysfunction in 96% of adults with proven food allergy and outperformed routine histology for diagnostic sensitivity [249]. Complementary immunostaining or electron microscopy of biopsies localizes loss or misdistribution of ZO1, occludin, or claudins and is routinely used to validate TEER/permeability findings, while circulating or luminal biomarkers, such as zonulin, IFABP, IL33, and TSLP reflect epithelial stress or loss and increasingly guide clinical trials that aim to restore barrier integrity.

Electrical impedance–based approaches are now translating these concepts directly to the clinic. On the skin, portable electrical impedance spectroscopy (EIS) probes quantify frequency‐dependent impedance [250, 251]; a 2024 controlled study showed EIS detects subclinical barrier impairment in AD and is less affected by ambient factors than transepidermal water loss, positioning it for therapy monitoring in dermatology clinics [252]. Within the esophagus, catheter or endoscope‐mounted mucosal impedance sensors provide an instantaneous readout of ionic permeability: baseline values fall two to three‐fold in reflux disease and EoE, predict response to proton pump inhibitors, and help differentiate functional heartburn from nonerosive reflux when pHmetry is equivocal [253]. Together, TEER/tracer assays, CLE‐guided imaging, molecular biomarkers, and emerging EIS‐based tools form a complementary toolkit that can interrogate epithelial barrier status across organs, from bench to bedside.

## Challenges and future directions

The epithelial barrier theory offers a valuable framework to understand and explain the rapid increase in the prevalence of chronic NCDs during the last six decades. Research in this area has been focused on the potential role of environmental factors interacting with host susceptibility, immune response, and the microbiome. However, establishing definitive causality and understanding the precise contribution of barrier dysfunction across the diverse spectrum of NCDs remain challenging. Rigorous research is needed to move beyond association and demonstrate causation in humans, particularly through longitudinal studies. Accurately assessing real‐world human exposure levels to potentially harmful substances (like detergents, emulsifiers, micro‐nanoplastics, and pollutants) and defining clear dose–response relationships are critical.

Furthermore, NCDs are inherently multifactorial; epithelial barrier dysfunction and microbiome changes must be considered alongside genetics, broader dietary patterns, lifestyle, and socioeconomic factors, stress, physical activity levels, and other environmental influences. Future research should focus on developing reliable biomarkers for epithelial barrier integrity, microbiome changes, immune system activation, and relevant environmental exposures, refining experimental models to better reflect human physiology and exposure scenarios, and exploring the complex feedback loops between the three main pillars: the epithelial barrier, the microbiome, and the immune system. Intervention studies targeting barrier protection or restoration (through lifestyle changes, specific dietary components, or novel therapeutics) and microbiome modulation are warranted.

In conclusion, there is substantial evidence from epidemiological, clinical, and experimental studies suggesting that compromised epithelial barriers are associated with a wide range of chronic inflammatory diseases. Substances common in modern environments appear capable of damaging these barriers, potentially initiating or exacerbating pathological processes involving microbial dysbiosis, immune dysregulation, and chronic inflammation. While the hypothesis requires further validation and refinement, particularly regarding direct causality and the relative importance of barrier dysfunction across different NCDs, it highlights the critical role of epithelial integrity in health maintenance. This perspective encourages greater scrutiny of the chemicals used in daily life, may stimulate the development of safer alternatives, and supports investigation of preventive and therapeutic strategies focused on preserving or restoring epithelial barrier function through lifestyle, diet, and potentially microbiome‐targeted interventions.

## Conflict of interest

R.D. is a cofounder and CEO of Seed Health. K.C.N. reports grants from the National Institute of Allergy and Infectious Diseases (NIAID), the National Heart, Lung, and Blood Institute (NHLBI), the National Institute of Environmental Health Sciences (NIEHS), the Immune Tolerance Network (ITN), and the National Institutes of Health (NIH) clinical research centers during the course of the study; in addition, other grants were received from IgGenix, Seed Health, ClostraBio, Cour, Alladapt, Excellergy, Red Tree Ventures, Regeneron, and Latitude outside the submitted work. In addition, K.C.N. has the following patents: ‘Mixed allergen composition and methods for using the same’, ‘Granulocyte‐based methods for detecting and monitoring immune system disorders’, and ‘Methods and Assays for Detecting and Quantifying Pure Subpopulations of White Blood Cells in Immune System Disorders’. M.A. has received research grants from the Swiss National Science Foundation, Bern; research grants from Stanford University; Leading House for the Latin American Region; and Seed Money Grants. She is a Scientific Advisory Board member of Stanford University Sean Parker Asthma Allergy Center, CA; an Advisory Board member of the LEO Foundation Skin Immunology Research Center, Copenhagen; and a Scientific Co‐Chair of the World Allergy Congress (WAC) Istanbul, 2022, Scientific Programme Committee Chair, EAACI. C.A.A. has received research grants from the Swiss National Science Foundation, European Union (EU CURE, EU Syn‐Air‐G), Novartis Research Institutes (Basel, Switzerland), Stanford University (Redwood City, California), Seed Health (Boston, USA), AO Research Institute (Davos, Switzerland) and SciBase (Stockholm, Sweden). He is the Co‐Chair for the EAACI Guidelines on Environmental Science in Allergic Diseases and Asthma; Chair of the EAACI Epithelial Cell Biology Working Group. He serves on the Advisory Boards of Sanofi/Regeneron (Bern, Switzerland, New York, USA), Stanford University Sean Parker Asthma Allergy Center (CA, USA), Novartis (Basel, Switzerland), GlaxoSmithKline (Zurich, Switzerland), Bristol‐Myers Squibb (New York, USA), Seed Health (Boston, USA), and SciBase (Stockholm, Sweden). C.A.A. is the Editor‐in‐Chief of Allergy. C.Z., H.B., I.O., S.A., Y.P., D.Y., B.Z., L.C., X.L., P.D., M.L., C.B., F.H.K.B., and M.‐C.B. declare no relevant conflicts of interest.

## Author contributions

C.Z., H.B., I.O., S.A., Y.P., D.Y., B.Z., L.C., X.L., P.D., M.L., C.B., F.H.K.B., R.D., K.C.N., M.‐C.B., M.A., and C.A.A. contributed to the conception, structure, development, writing, and review of this manuscript.

## References

[1] Akdis CA (2021) Does the epithelial barrier hypothesis explain the increase in allergy, autoimmunity and other chronic conditions? Nat Rev Immunol 21, 739–751. 33846604 10.1038/s41577-021-00538-7

[2] Trautmann A , Akdis M , Kleemann D , Altznauer F , Simon HU , Graeve T , Noll M , Bröcker EB , Blaser K and Akdis CA (2000) T cell–mediated Fas‐induced keratinocyte apoptosis plays a key pathogenetic role in eczematous dermatitis. J Clin Invest 106, 25–35. 10880045 10.1172/JCI9199 PMC517909

[3] Trautmann A , Schmid‐Grendelmeier P , Krüger K , Crameri R , Akdis M , Akkaya A , Bröcker EB , Blaser K and Akdis CA (2002) T cells and eosinophils cooperate in the induction of bronchial epithelial cell apoptosis in asthma. J Allergy Clin Immunol 109, 329–337. 11842305 10.1067/mai.2002.121460

[4] Basinski TM , Holzmann D , Eiwegger T , Zimmermann M , Klunker S , Meyer N , Schmid‐Grendelmeier P , Jutel M and Akdis CA (2009) Dual nature of T cell–epithelium interaction in chronic rhinosinusitis. J Allergy Clin Immunol 124, 74–80.e8. 19523671 10.1016/j.jaci.2009.04.019

[5] Akdis CA (2006) Allergy and hypersensitivity: mechanisms of allergic disease. Curr Opin Immunol 18, 718–726. 17029937 10.1016/j.coi.2006.09.016

[6] Akdis M (2006) Healthy immune response to allergens: T regulatory cells and more. Curr Opin Immunol 18, 738–744. 17023149 10.1016/j.coi.2006.06.003

[7] Chang X , Puddicombe SM , Field SA , Haywood J , Broughton‐Head V , Puxeddu I , Haitchi HM , Vernon‐Wilson E , Sammut D , Bedke N *et al*. (2011) Defective epithelial barrier function in asthma. J Allergy Clin Immunol 128, 549–556.e12. 21752437 10.1016/j.jaci.2011.05.038

[8] Soyka MB , Wawrzyniak P , Eiwegger T , Holzmann D , Treis A , Wanke K , Kast JI and Akdis CA (2012) Defective epithelial barrier in chronic rhinosinusitis: the regulation of tight junctions by IFN‐γ and IL‐4. J Allergy Clin Immunol 130, 1087–1096.e10. 22840853 10.1016/j.jaci.2012.05.052

[9] Benedetto AD , Rafaels N , McGirt LY , Ivanov AI , Georas SN , Cheadle C , Berger AE , Zhang K , Vidyasagar S , Yoshida T *et al*. (2011) Tight junction defects in patients with atopic dermatitis. J Allergy Clin Immunol 127, 773–786.e7. 21163515 10.1016/j.jaci.2010.10.018 PMC3049863

[10] Irvine AD , McLean WHI and Leung DYM (2011) Filaggrin mutations associated with skin and allergic diseases. N Engl J Med 365, 1315–1327. 21991953 10.1056/NEJMra1011040

[11] Ferreira MA , Vonk JM , Baurecht H , Marenholz I , Tian C , Hoffman JD , Helmer Q , Tillander A , Ullemar V , van Dongen J *et al*. (2017) Shared genetic origin of asthma, hay fever and eczema elucidates allergic disease biology. Nat Genet 49, 1752–1757. 29083406 10.1038/ng.3985 PMC5989923

[12] Wawrzyniak P , Wawrzyniak M , Wanke K , Sokolowska M , Bendelja K , Rückert B , Globinska A , Jakiela B , Kast JI , Idzko M *et al*. (2017) Regulation of bronchial epithelial barrier integrity by type 2 cytokines and histone deacetylases in asthmatic patients. J Allergy Clin Immunol 139, 93–103. 27312821 10.1016/j.jaci.2016.03.050

[13] Sugita K , Altunbulakli C , Morita H , Sugita A , Kubo T , Kimura R , Goto H , Yamamoto O , Rückert B , Akdis M *et al*. (2019) Human type 2 innate lymphoid cells disrupt skin keratinocyte tight junction barrier by IL‐13. Allergy 74, 2534–2537. 31166013 10.1111/all.13935

[14] Xian M , Wawrzyniak P , Rückert B , Duan S , Meng Y , Sokolowska M , Globinska A , Zhang L , Akdis M and Akdis CA (2016) Anionic surfactants and commercial detergents decrease tight junction barrier integrity in human keratinocytes. J Allergy Clin Immunol 138, 890–893.e9. 27596709 10.1016/j.jaci.2016.07.003

[15] Altunbulakli C , Reiger M , Neumann AU , Garzorz‐Stark N , Fleming M , Huelpuesch C , Castro‐Giner F , Eyerich K , Akdis CA and Traidl‐Hoffmann C (2018) Relations between epidermal barrier dysregulation and *Staphylococcus* species–dominated microbiome dysbiosis in patients with atopic dermatitis. J Allergy Clin Immunol 142, 1643–1647.e12. 30048670 10.1016/j.jaci.2018.07.005

[16] Wang M , Tan G , Eljaszewicz A , Meng Y , Wawrzyniak P , Acharya S , Altunbulakli C , Westermann P , Dreher A , Yan L *et al*. (2019) Laundry detergents and detergent residue after rinsing directly disrupt tight junction barrier integrity in human bronchial epithelial cells. J Allergy Clin Immunol 143, 1892–1903. 30500342 10.1016/j.jaci.2018.11.016

[17] Xian M , Ma S , Wang K , Lou H , Wang Y , Zhang L , Wang C and Akdis CA (2020) Particulate matter 2.5 causes deficiency in barrier integrity in human nasal epithelial cells. Allergy, Asthma Immunol Res 12, 56–71. 31743964 10.4168/aair.2020.12.1.56 PMC6875480

[18] Michaudel C , Mackowiak C , Maillet I , Fauconnier L , Akdis CA , Sokolowska M , Dreher A , Tan HTT , Quesniaux VF , Ryffel B *et al*. (2018) Ozone exposure induces respiratory barrier biphasic injury and inflammation controlled by IL‐33. J Allergy Clin Immunol 142, 942–958. 29331644 10.1016/j.jaci.2017.11.044

[19] Jin Y , Liang L , Tu W , Luo T and Fu Z (2019) Impacts of polystyrene microplastic on the gut barrier, microbiota and metabolism of mice. Sci Total Environ 649, 308–317. 30176444 10.1016/j.scitotenv.2018.08.353

[20] Ogulur I , Pat Y , Aydin T , Yazici D , Rückert B , Peng Y , Kim J , Radzikowska U , Westermann P , Sokolowska M *et al*. (2023) Gut epithelial barrier damage caused by dishwasher detergents and rinse aids. J Allergy Clin Immunol 151, 469–484. 36464527 10.1016/j.jaci.2022.10.020

[21] Ogulur I , Yazici D , Pat Y , Bingöl EN , Babayev H , Ardicli S , Heider A , Rückert B , Sampath V , Dhir R *et al*. (2023) Mechanisms of gut epithelial barrier impairment caused by food emulsifiers polysorbate 20 and polysorbate 80. Allergy 78, 2441–2455. 37530764 10.1111/all.15825

[22] Rinaldi AO , Li M , Barletta E , D'Avino P , Yazici D , Pat Y , Ward S , Burla D , Tan G , Askary N *et al*. (2024) Household laundry detergents disrupt barrier integrity and induce inflammation in mouse and human skin. Allergy 79, 128–141. 37766519 10.1111/all.15891

[23] Sun N , Ogulur I , Mitamura Y , Yazici D , Pat Y , Bu X , Li M , Zhu X , Babayev H , Ardicli S *et al*. (2024) The epithelial barrier theory and its associated diseases. Allergy 79, 3192–3237. 39370939 10.1111/all.16318 PMC11657050

[24] Tajik N , Frech M , Schulz O , Schälter F , Lucas S , Azizov V , Dürholz K , Steffen F , Omata Y , Rings A *et al*. (2020) Targeting Zonulin and intestinal epithelial barrier function to prevent onset of arthritis. Nat Commun 11, 1995. 32332732 10.1038/s41467-020-15831-7 PMC7181728

[25] Cortese A , Lova L , Comoli P , Volpe E , Villa S , Mallucci G , la Salvia S , Romani A , Franciotta D , Bollati V *et al*. (2020) Air pollution as a contributor to the inflammatory activity of multiple sclerosis. J Neuroinflammation 17, 334. 33158438 10.1186/s12974-020-01977-0 PMC7645903

[26] Cerqueira É , Marinho DA , Neiva HP and Lourenço O (2020) Inflammatory effects of high and moderate intensity exercise—a systematic review. Front Physiol 10, 1550. 31992987 10.3389/fphys.2019.01550 PMC6962351

[27] Kistler W , Villiger M , Villiger B , Yazici D , Pat Y , Mitamura Y , Ardicli S , Skolnick S , Dhir R , Akdis M *et al*. (2024) Epithelial barrier theory in the context of nutrition and environmental exposure in athletes. Allergy 79, 2912–2923. 39011970 10.1111/all.16221

[28] El Assar M , Álvarez‐Bustos A , Sosa P , Angulo J and Rodríguez‐Mañas L (2022) Effect of physical activity/exercise on oxidative stress and inflammation in muscle and vascular aging. Int J Mol Sci 23, 8713. 35955849 10.3390/ijms23158713 PMC9369066

[29] Allen JM , Mailing LJ , Cohrs J , Salmonson C , Fryer JD , Nehra V , Hale VL , Kashyap P , White BA and Woods JA (2018) Exercise training‐induced modification of the gut microbiota persists after microbiota colonization and attenuates the response to chemically‐induced colitis in gnotobiotic mice. Gut Microbes 9, 115–130. 28862530 10.1080/19490976.2017.1372077 PMC5989796

[30] Yu C , Liu S , Niu Y and Fu L (2022) Exercise protects intestinal epithelial barrier from high fat diet‐ induced permeabilization through SESN2/AMPKα1/HIF‐1α signaling. J Nutr Biochem 107, 109059. 35643285 10.1016/j.jnutbio.2022.109059

[31] Motiani KK , Collado MC , Eskelinen JJ , Virtanen KA , Löyttyniemi E , Salminen S , Nuutila P , Kalliokoski KK and Hannukainen JC (2020) Exercise training modulates gut microbiota profile and improves endotoxemia. Med Sci Sports Exerc 52, 94–104. 31425383 10.1249/MSS.0000000000002112 PMC7028471

[32] Estaki M , Pither J , Baumeister P , Little JP , Gill SK , Ghosh S , Ahmadi‐Vand Z , Marsden KR and Gibson DL (2016) Cardiorespiratory fitness as a predictor of intestinal microbial diversity and distinct metagenomic functions. Microbiome 4, 1–13. 27502158 10.1186/s40168-016-0189-7 PMC4976518

[33] Lira FS , Rosa JC , Pimentel GD , Souza HA , Caperuto EC , Carnevali LC Jr , Seelaender M , Damaso AR , Oyama LM , de Mello MT *et al*. (2010) Endotoxin levels correlate positively with a sedentary lifestyle and negatively with highly trained subjects. Lipids Health Dis 9, 82. 20684772 10.1186/1476-511X-9-82 PMC2922209

[34] Powers SK , Deminice R , Ozdemir M , Yoshihara T , Bomkamp MP and Hyatt H (2020) Exercise‐induced oxidative stress: friend or foe? J Sport Health Sci 9, 415–425. 32380253 10.1016/j.jshs.2020.04.001 PMC7498668

[35] Tian D and Meng J (2019) Exercise for prevention and relief of cardiovascular disease: prognoses, mechanisms, and approaches. Oxidative Med Cell Longev 2019, 3756750. 10.1155/2019/3756750 PMC6481017 31093312

[36] Gleeson M and Pyne DB (2016) Respiratory inflammation and infections in high‐performance athletes. Immunol Cell Biol 94, 124–131. 26568028 10.1038/icb.2015.100 PMC7165758

[37] Walsh NP and Oliver SJ (2016) Exercise, immune function and respiratory infection: an update on the influence of training and environmental stress. Immunol Cell Biol 94, 132–139. 26563736 10.1038/icb.2015.99

[38] Rehm K , Sunesara I and Marshall GD (2015) Increased circulating anti‐inflammatory cells in marathon‐trained runners. Int J Sports Med 94, 832–836. 10.1055/s-0035-1547218 26038877

[39] Forsyth A and Mantzioris E (2023) An online exploratory survey of Australian athletes' and exercisers' use of and attitudes towards ultra‐processed sports foods. Br J Nutr 130, 1625–1636. 36999372 10.1017/S0007114523000648 PMC10551470

[40] Mårtensson S , Nordebo K and Malm C (2014) High training volumes are associated with a low number of self‐reported sick days in elite endurance athletes. J Sports Sci Med 13, 929–933. 25435787 PMC4234964

[41] Ardicli S , Ardicli O , Yazici D , Pat Y , Babayev H , Xiong P , Zeyneloglu C , Garcia‐Sanchez A , Shi LL , Viscardi OG *et al*. (2024) Epithelial barrier dysfunction and associated diseases in companion animals: differences and similarities between humans and animals and research needs. Allergy 79, 3238–3268. 39417247 10.1111/all.16343 PMC11657079

[42] Jutel M , Mosnaim GS , Bernstein JA , del Giacco S , Khan DA , Nadeau KC , Pali‐Schöll I , Torres MJ , Zemelka‐Wiacek M and Agache I (2023) The one health approach for allergic diseases and asthma. Allergy 78, 1777–1793. 37119496 10.1111/all.15755

[43] Pali‐Schöll I , Roth‐Walter F and Jensen‐Jarolim E (2021) One health in allergology: a concept that connects humans, animals, plants, and the environment. Allergy 76, 2630–2633. 33665860 10.1111/all.14804 PMC8359833

[44] O'Neill DG , James H , Brodbelt DC , Church DB and Pegram C (2021) Prevalence of commonly diagnosed disorders in UK dogs under primary veterinary care: results and applications. BMC Vet Res 17, 69. 33593363 10.1186/s12917-021-02775-3 PMC7888168

[45] Hardy J and Gajanayake I (2022) Diagnosis and management of adverse food reactions in dogs and cats. In Pract 44, 196–203.

[46] Bertero A , Fossati P and Caloni F (2020) Indoor poisoning of companion animals by chemicals. Sci Total Environ 733, 139366. 32446086 10.1016/j.scitotenv.2020.139366

[47] Knapp DW , Peer WA , Conteh A , Diggs AR , Cooper BR , Glickman NW , Bonney PL , Stewart JC , Glickman LT and Murphy AS (2013) Detection of herbicides in the urine of pet dogs following home lawn chemical application. Sci Total Environ 456‐457, 34–41. 10.1016/j.scitotenv.2013.03.019 23584031

[48] Pawankar R and Akdis CA (2023) Climate change and the epithelial barrier theory in allergic diseases: a one health approach to a green environment. Allergy 78, 2829–2834. 37675628 10.1111/all.15885

[49] Kogawa AC , Cernic BG , do Couto LGD and Salgado HRN (2017) Synthetic detergents: 100 years of history. Saudi Pharm J 25, 934–938. 28951681 10.1016/j.jsps.2017.02.006 PMC5605839

[50] Leoty‐Okombi S , Gillaizeau F , Leuillet S , Douillard B , le Fresne‐Languille S , Carton T , de Martino A , Moussou P , Bonnaud‐Rosaye C and André V (2021) Effect of sodium lauryl sulfate (SLS) applied as a patch on human skin physiology and its microbiota. Cosmetics 8, 6.

[51] Wilhelm KP , Freitag G and Wolff HH (1994) Surfactant‐induced skin irritation and skin repair: evaluation of the acute human irritation model by noninvasive techniques. J Am Acad Dermatol 30, 944–949. 8188884 10.1016/s0190-9622(94)70114-8

[52] Douwes J , Slater T , Shanthakumar M , McLean D , Firestone RT , Judd L and Pearce N (2017) Determinants of hand dermatitis, urticaria and loss of skin barrier function in professional cleaners in New Zealand. Int J Occup Environ Health 23, 110–119. 29359638 10.1080/10773525.2018.1427307 PMC6060852

[53] Viennois E and Chassaing B (2018) First victim, later aggressor: how the intestinal microbiota drives the pro‐inflammatory effects of dietary emulsifiers? Gut Microbes 9, 1–4. 10.1080/19490976.2017.1421885 PMC6219590 29437527

[54] Chassaing B , Koren O , Goodrich JK , Poole AC , Srinivasan S , Ley RE and Gewirtz AT (2015) Dietary emulsifiers impact the mouse gut microbiota promoting colitis and metabolic syndrome. Nature 519, 92–96. 25731162 10.1038/nature14232 PMC4910713

[55] Gullikson GW , Cline WS , Lorenzsonn V , Benz L , Olsen WA and Bass P (1977) Effects of anionic surfactants on hamster small intestinal membrane structure and function: relationship to surface activity. Gastroenterology 73, 501–511. 892348

[56] Brenchley JM and Douek DC (2012) Microbial translocation across the GI tract. Annu Rev Immunol 30, 149–173. 22224779 10.1146/annurev-immunol-020711-075001 PMC3513328

[57] Doyle AD , Masuda MY , Pyon GC , Luo H , Putikova A , LeSuer WE , Flashner S , Rank MA , Nakagawa H , Kita H *et al*. (2023) Detergent exposure induces epithelial barrier dysfunction and eosinophilic inflammation in the esophagus. Allergy 78, 192–201. 35899466 10.1111/all.15457 PMC9797443

[58] Stock V , Böhmert L , Lisicki E , Block R , Cara‐Carmona J , Pack LK , Selb R , Lichtenstein D , Voss L , Henderson CJ *et al*. (2019) Uptake and effects of orally ingested polystyrene microplastic particles in vitro and in vivo. Arch Toxicol 93, 1817–1833. doi: 10.1007/s00204-019-02478-7 31139862

[59] Hollóczki O and Gehrke S (2020) Can nanoplastics alter cell membranes? ChemPhysChem 21, 9–12. 31483076 10.1002/cphc.201900481 PMC6973106

[60] Hollóczki O and Gehrke S (2019) Nanoplastics can change the secondary structure of proteins. Sci Rep 9, 16013. 31690820 10.1038/s41598-019-52495-w PMC6831684

[61] Mitamura Y , Ogulur I , Pat Y , Rinaldi AO , Ardicli O , Cevhertas L , Brüggen MC , Traidl‐Hoffmann C , Akdis M and Akdis CA (2021) Dysregulation of the epithelial barrier by environmental and other exogenous factors. Contact Derm 85, 615–626. 10.1111/cod.13959 PMC9293165 34420214

[62] Qin L , Yang L , Liu L , Tong S , Liu Q , Li G , Zhang H , Zhu WY , Liu G , Zheng M *et al*. (2024) Oxidative potential and persistent free radicals in dust storm particles and their associations with hospitalization. Nat Commun 15, 10827. 39738021 10.1038/s41467-024-55151-8 PMC11685391

[63] Tirichen H , Yaigoub H , Xu W , Wu C , Li R and Li Y (2021) Mitochondrial reactive oxygen species and their contribution in chronic kidney disease progression through oxidative stress. Front Physiol 12, 627837. 33967820 10.3389/fphys.2021.627837 PMC8103168

[64] Bhatti JS , Bhatti GK and Reddy PH (2017) Mitochondrial dysfunction and oxidative stress in metabolic disorders — a step towards mitochondria based therapeutic strategies. Biochim Biophys Acta 1863, 1066–1077. 10.1016/j.bbadis.2016.11.010 PMC5423868 27836629

[65] Palma FR , Gantner BN , Sakiyama MJ , Kayzuka C , Shukla S , Lacchini R , Cunniff B and Bonini MG (2024) ROS production by mitochondria: function or dysfunction? Oncogene 43, 295–303. 38081963 10.1038/s41388-023-02907-z

[66] Suda K , Moriyama Y , Razali N , Chiu Y , Masukagami Y , Nishimura K , Barbee H , Takase H , Sugiyama S , Yamazaki Y *et al*. (2024) Plasma membrane damage limits replicative lifespan in yeast and induces premature senescence in human fibroblasts. Nat Aging 4, 319–335. doi: 10.1038/s43587-024-00575-6 38388781 PMC10950784

[67] Peoples JN , Saraf A , Ghazal N , Pham TT and Kwong JQ (2019) Mitochondrial dysfunction and oxidative stress in heart disease. Exp Mol Med 51, 1–13. 10.1038/s12276-019-0355-7 PMC6923355 31857574

[68] Khan TA , Mahler HC and Kishore RSK (2015) Key interactions of surfactants in therapeutic protein formulations: a review. Eur J Pharm Biopharm 97(Pt A), 60–67. 26435336 10.1016/j.ejpb.2015.09.016

[69] He L , Zhang L , Meng F , Wei J , Chen F , Qin S , Jin G and Cao H (2025) Dietary emulsifier polysorbate 80‐induced lipotoxicity promotes intestinal senescence. Food Res Int 209, 116165. 40253120 10.1016/j.foodres.2025.116165

[70] Trifunovic A , Wredenberg A , Falkenberg M , Spelbrink JN , Rovio AT , Bruder CE , Bohlooly‐Y M , Gidlöf S , Oldfors A , Wibom R *et al*. (2004) Premature ageing in mice expressing defective mitochondrial DNA polymerase. Nature 429, 417–423. 15164064 10.1038/nature02517

[71] Zong Y , Li H , Liao P , Chen L , Pan Y , Zheng Y , Zhang C , Liu D , Zheng M and Gao J (2024) Mitochondrial dysfunction: mechanisms and advances in therapy. Signal Transduct Target Ther 9, 1–29. 38744846 10.1038/s41392-024-01839-8 PMC11094169

[72] Costa R , Peruzzo R , Bachmann M , Montà GD , Vicario M , Santinon G , Mattarei A , Moro E , Quintana‐Cabrera R , Scorrano L *et al*. (2019) Impaired mitochondrial ATP production downregulates Wnt signaling via ER stress induction. Cell Rep 28, 1949–1960.e6. 31433973 10.1016/j.celrep.2019.07.050

[73] Schütt F , Aretz S , Auffarth GU and Kopitz J (2012) Moderately reduced ATP levels promote oxidative stress and debilitate autophagic and phagocytic capacities in human RPE cells. Invest Ophthalmol Vis Sci 53, 5354–5361. 22789922 10.1167/iovs.12-9845

[74] Ma Z , Wei Q , Dong G , Huo Y and Dong Z (2014) DNA damage response in renal ischemia–reperfusion and ATP‐depletion injury of renal tubular cells. Biochim Biophys Acta 1842, 1088–1096. 24726884 10.1016/j.bbadis.2014.04.002 PMC4038345

[75] Hayashi R , Okumura H , Isono M , Yamauchi M , Unami D , Lusi RT , Yamamoto M , Kato Y , Uchihara Y and Shibata A (2024) Inhibition of intracellular ATP synthesis impairs the recruitment of homologous recombination factors after ionizing radiation. J Radiat Res (Tokyo) 65, 263–271. 38461549 10.1093/jrr/rrae005 PMC11115441

[76] Daverkausen‐Fischer L and Pröls F (2022) Regulation of calcium homeostasis and flux between the endoplasmic reticulum and the cytosol. J Biol Chem 298, 102061. 35609712 10.1016/j.jbc.2022.102061 PMC9218512

[77] Brookes PS , Yoon Y , Robotham JL , Anders MW and Sheu SS (2004) Calcium, ATP, and ROS: a mitochondrial love‐hate triangle. Am J Physiol‐Cell Physiol 287, C817–C833. 15355853 10.1152/ajpcell.00139.2004

[78] Marchi S , Patergnani S and Pinton P (2014) The endoplasmic reticulum‐mitochondria connection: one touch, multiple functions. Biochim Biophys Acta 1837, 461–469. 24211533 10.1016/j.bbabio.2013.10.015

[79] Liu X , Kim CN , Yang J , Jemmerson R and Wang X (1996) Induction of apoptotic program in cell‐free extracts: requirement for dATP and cytochrome c. Cell 86, 147–157. 8689682 10.1016/s0092-8674(00)80085-9

[80] Vitale I , Pietrocola F , Guilbaud E , Aaronson SA , Abrams JM , Adam D , Agostini M , Agostinis P , Alnemri ES , Altucci L *et al*. (2023) Apoptotic cell death in disease—current understanding of the NCCD 2023. Cell Death Differ 30, 1097–1154. 37100955 10.1038/s41418-023-01153-w PMC10130819

[81] de Almeida AJPO , de Oliveira JCPL , da Silva Pontes LV , de Souza Júnior JF , Gonçalves TAF , Dantas SH , de Almeida Feitosa MS , Silva AO and de Medeiros IA (2022) ROS: basic concepts, sources, cellular signaling, and its implications in aging pathways. Oxidative Med Cell Longev 2022, 1225578. 10.1155/2022/1225578 PMC9605829 36312897

[82] Nie B , Liu X , Lei C , Liang X , Zhang D and Zhang J (2024) The role of lysosomes in airborne particulate matter‐induced pulmonary toxicity. Sci Total Environ 919, 170893. 38342450 10.1016/j.scitotenv.2024.170893

[83] Wei M , Bao G , Li S , Yang Z , Cheng C and Le W (2022) PM2.5 exposure triggers cell death through lysosomal membrane permeabilization and leads to ferroptosis insensitivity via the autophagy dysfunction/p62‐KEAP1‐NRF2 activation in neuronal cells. Ecotoxicol Environ Saf 248, 114333. 36446170 10.1016/j.ecoenv.2022.114333

[84] Sies H , Belousov VV , Chandel NS , Davies MJ , Jones DP , Mann GE , Murphy MP , Yamamoto M and Winterbourn C (2022) Defining roles of specific reactive oxygen species (ROS) in cell biology and physiology. Nat Rev Mol Cell Biol 23, 499–515. doi: 10.1038/s41580-022-00456-z 35190722

[85] Glasauer A and Chandel NS (2013) ROS. Curr Biol 23, R100–R102. doi: 10.1016/j.cub.2012.12.011 23391379

[86] Saito K , Orimo K , Kubo T , Tamari M , Yamada A , Motomura K , Sugiyama H , Matsuoka R , Nagano N , Hayashi Y *et al*. (2023) Laundry detergents and surfactants‐induced eosinophilic airway inflammation by increasing IL‐33 expression and activating ILC2s. Allergy 78, 1878–1892. 37163231 10.1111/all.15762

[87] Barbier E , Carpentier J , Simonin O , Gosset P , Platel A , Happillon M , Alleman LY , Perdrix E , Riffault V , Chassat T *et al*. (2023) Oxidative stress and inflammation induced by air pollution‐derived PM2.5 persist in the lungs of mice after cessation of their sub‐chronic exposure. Environ Int 181, 108248. 37857188 10.1016/j.envint.2023.108248

[88] Liu S , Li L , Liu S , Liu L , Xiao X , Zhou D , Zhu C and She X (2024) Reactive oxygen species‐induced microplastics aging: implications for environmental fate and ecological impact. TrAC Trends Anal Chem 173, 117648.

[89] Gaschler MM and Stockwell BR (2017) Lipid peroxidation in cell death. Biochem Biophys Res Commun 482, 419–425. 28212725 10.1016/j.bbrc.2016.10.086 PMC5319403

[90] Hirata Y , Cai R , Volchuk A , Steinberg BE , Saito Y , Matsuzawa A , Grinstein S and Freeman SA (2023) Lipid peroxidation increases membrane tension, Piezo1 gating, and cation permeability to execute ferroptosis. Curr Biol 33, 1282–1294.e5. 36898371 10.1016/j.cub.2023.02.060

[91] Ayala A , Muñoz MF and Argüelles S (2014) Lipid peroxidation: production, metabolism, and signaling mechanisms of malondialdehyde and 4‐Hydroxy‐2‐Nonenal. Oxidative Med Cell Longev 2014, 360438. 10.1155/2014/360438 PMC4066722 24999379

[92] Lee JY , Kim WK , Bae KH , Lee SC and Lee EW (2021) Lipid metabolism and ferroptosis. Biology 10, 184. 33801564 10.3390/biology10030184 PMC8000263

[93] Yan HF , Xu S , Li H , Belaidi AA and Lei P (2021) Ferroptosis: mechanisms and links with diseases. Signal Transduct Target Ther 6, 1–16. 33536413 10.1038/s41392-020-00428-9 PMC7858612

[94] von Krusenstiern AN , Robson RN , Qian N , Qiu B , Hu F , Reznik E , Smith N , Zandkarimi F , Estes VM , Dupont M *et al*. (2023) Identification of essential sites of lipid peroxidation in ferroptosis. Nat Chem Biol 19, 719–730. 36747055 10.1038/s41589-022-01249-3 PMC10238648

[95] Mu Y , Sun J , Li Z , Zhang W , Liu Z , Li C , Peng C , Cui G , Shao H and du Z (2022) Activation of pyroptosis and ferroptosis is involved in the hepatotoxicity induced by polystyrene microplastics in mice. Chemosphere 291, 132944. 34793849 10.1016/j.chemosphere.2021.132944

[96] Park M , Park S , Choi Y , Cho YL , Kim MJ , Park YJ , Chung SW , Lee H and Lee SJ (2024) The mechanism underlying correlation of particulate matter‐induced ferroptosis with inflammasome activation and iron accumulation in macrophages. Cell Death Discov 10, 1–11. 38491062 10.1038/s41420-024-01874-y PMC10943117

[97] Hacioglu C (2024) Long‐term exposure of sucralose induces neuroinflammation and ferroptosis in human microglia cells via SIRT1/NLRP3/IL‐1β/GPx4 signaling pathways. Food Sci Nutr 12, 9094–9107. 39619997 10.1002/fsn3.4488 PMC11606902

[98] Dixon SJ and Olzmann JA (2024) The cell biology of ferroptosis. Nat Rev Mol Cell Biol 25, 424–442. 38366038 10.1038/s41580-024-00703-5 PMC12187608

[99] Chen X , Shi C , He M , Xiong S and Xia X (2023) Endoplasmic reticulum stress: molecular mechanism and therapeutic targets. Signal Transduct Target Ther 8, 1–40. 37709773 10.1038/s41392-023-01570-w PMC10502142

[100] Almanza A , Carlesso A , Chintha C , Creedican S , Doultsinos D , Leuzzi B , Luís A , McCarthy N , Montibeller L , More S *et al*. (2019) Endoplasmic reticulum stress signalling – from basic mechanisms to clinical applications. FEBS J 286, 241–278. 30027602 10.1111/febs.14608 PMC7379631

[101] Malhotra JD and Kaufman RJ (2007) Endoplasmic reticulum stress and oxidative stress: a vicious cycle or a double‐edged sword? Antioxid Redox Signal 9, 2277–2294. 17979528 10.1089/ars.2007.1782

[102] Cao SS and Kaufman RJ (2014) Endoplasmic reticulum stress and oxidative stress in cell fate decision and human disease. Antioxid Redox Signal 21, 396–413. 24702237 10.1089/ars.2014.5851 PMC4076992

[103] Hetz C (2012) The unfolded protein response: controlling cell fate decisions under ER stress and beyond. Nat Rev Mol Cell Biol 13, 89–102. 22251901 10.1038/nrm3270

[104] Park S , Sethi S and Bouret SG (2019) Non‐nutritive sweeteners induce hypothalamic ER stress causing abnormal axon outgrowth. Front Endocrinol 10, 876. 10.3389/fendo.2019.00876 PMC6928131 31920985

[105] Li G , Scull C , Ozcan L and Tabas I (2010) NADPH oxidase links endoplasmic reticulum stress, oxidative stress, and PKR activation to induce apoptosis. J Cell Biol 191, 1113–1125. 21135141 10.1083/jcb.201006121 PMC3002036

[106] Haynes CM , Titus EA and Cooper AA (2004) Degradation of misfolded proteins prevents ER‐derived oxidative stress and cell death. Mol Cell 15, 767–776. 15350220 10.1016/j.molcel.2004.08.025

[107] Bhattarai KR , Riaz TA , Kim HR and Chae HJ (2021) The aftermath of the interplay between the endoplasmic reticulum stress response and redox signaling. Exp Mol Med 53, 151–167. 33558590 10.1038/s12276-021-00560-8 PMC8080639

[108] Fun XH and Thibault G (2020) Lipid bilayer stress and proteotoxic stress‐induced unfolded protein response deploy divergent transcriptional and non‐transcriptional programmes. Biochim Biophys Acta 1865, 158449. 10.1016/j.bbalip.2019.04.009 31028913

[109] Halbleib K , Pesek K , Covino R , Hofbauer HF , Wunnicke D , Hänelt I , Hummer G and Ernst R (2017) Activation of the unfolded protein response by lipid bilayer stress. Mol Cell 67, 673–684.e8. 28689662 10.1016/j.molcel.2017.06.012

[110] Zhang M , Wang Y , Wong RMS , Yung KKL and Li R (2022) Fine particulate matter induces endoplasmic reticulum stress‐mediated apoptosis in human SH‐SY5Y cells. Neurotoxicology 88, 187–195. 34813867 10.1016/j.neuro.2021.11.012

[111] Ragusa A , Matta M , Cristiano L , Matassa R , Battaglione E , Svelato A , de Luca C , D'Avino S , Gulotta A , Rongioletti MCA *et al*. (2022) Deeply in Plasticenta: presence of microplastics in the intracellular compartment of human placentas. Int J Environ Res Public Health 19, 11593. 36141864 10.3390/ijerph191811593 PMC9517680

[112] Shukla N , Pomarico E , Hecht CJS , Taylor EA , Chergui M and Othon CM (2018) Hydrophobic interactions of sucralose with protein structures. Arch Biochem Biophys 639, 38–43. 29288052 10.1016/j.abb.2017.12.013

[113] Anand BG , Prajapati KP , Dubey K , Ahamad N , Shekhawat DS , Rath PC , Joseph GK and Kar K (2019) Self‐assembly of artificial sweetener aspartame yields amyloid‐like cytotoxic nanostructures. ACS Nano 13, 6033–6049. 31021591 10.1021/acsnano.9b02284

[114] Laing S , Wang G , Briazova T , Zhang C , Wang A , Zheng Z , Gow A , Chen AF , Rajagopalan S , Chen LC *et al*. (2010) Airborne particulate matter selectively activates endoplasmic reticulum stress response in the lung and liver tissues. Am J Physiol Cell Physiol 299, C736–C749. 20554909 10.1152/ajpcell.00529.2009 PMC2957267

[115] Wang F , Zhang Q , Cui J , Bao B , Deng X , Liu L and Guo MY (2023) Polystyrene microplastics induce endoplasmic reticulum stress, apoptosis and inflammation by disrupting the gut microbiota in carp intestines. Environ Pollut 323, 121233. 36804561 10.1016/j.envpol.2023.121233

[116] Averill‐Bates D (2024) Reactive oxygen species and cell signaling. Review. Biochim Biophys Acta Mol Cell Res 1871, 119573. 37949302 10.1016/j.bbamcr.2023.119573

[117] Mattie MD , McElwee MK and Freedman JH (2008) Mechanism of copper activated transcription: activation of AP‐1, and the JNK/SAPK and p38 signal transduction pathways. J Mol Biol 383, 1008–1018. 18793645 10.1016/j.jmb.2008.08.080 PMC2662727

[118] Gloire G , Legrand‐Poels S and Piette J (2006) NF‐kappaB activation by reactive oxygen species: fifteen years later. Biochem Pharmacol 72, 1493–1505. 16723122 10.1016/j.bcp.2006.04.011

[119] Morgan MJ and Liu Z g (2011) Crosstalk of reactive oxygen species and NF‐κB signaling. Cell Res 21, 103–115. 21187859 10.1038/cr.2010.178 PMC3193400

[120] Lebedev AV , Levitsky DO , Loginov VA and Smirnov VN (1982) The effect of primary products of lipid peroxidation on the transmembrane transport of calcium ions. J Mol Cell Cardiol 14, 99–103. 10.1016/0022-2828(82)90136-5 6815334

[121] Shields HJ , Traa A and Van Raamsdonk JM (2021) Beneficial and detrimental effects of reactive oxygen species on lifespan: a comprehensive review of comparative and experimental studies. Front Cell Dev Biol 9, 628157. 33644065 10.3389/fcell.2021.628157 PMC7905231

[122] Pham‐Huy LA , He H and Pham‐Huy C (2008) Free radicals, antioxidants in disease and health. Int J Biomed Sci 4, 89–96. 23675073 PMC3614697

[123] Wei X , Xie F , Zhou X , Wu Y , Yan H , Liu T , Huang J , Wang F , Zhou F and Zhang L (2022) Role of pyroptosis in inflammation and cancer. Cell Mol Immunol 19, 971–992. 35970871 10.1038/s41423-022-00905-x PMC9376585

[124] Ding J , Wang K , Liu W , She Y , Sun Q , Shi J , Sun H , Wang DC and Shao F (2016) Pore‐forming activity and structural autoinhibition of the gasdermin family. Nature 535, 111–116. 27281216 10.1038/nature18590

[125] Martinon F , Burns K and Tschopp J (2002) The Inflammasome: a molecular platform triggering activation of inflammatory caspases and processing of proIL‐β. Mol Cell 10, 417–426. 12191486 10.1016/s1097-2765(02)00599-3

[126] Liu Y , Pan R , Ouyang Y , Gu W , Xiao T , Yang H , Tang L , Wang H , Xiang B and Chen P (2024) Pyroptosis in health and disease: mechanisms, regulation and clinical perspective. Signal Transduct Target Ther 9, 1–28. 39300122 10.1038/s41392-024-01958-2 PMC11413206

[127] Schroder K and Tschopp J (2010) The inflammasomes. Cell 140, 821–832. 20303873 10.1016/j.cell.2010.01.040

[128] de Zoete MR , Palm NW , Zhu S and Flavell RA (2014) Inflammasomes. Cold Spring Harb Perspect Biol 6, a016287. 25324215 10.1101/cshperspect.a016287 PMC4292152

[129] Hornung V , Ablasser A , Charrel‐Dennis M , Bauernfeind F , Horvath G , Caffrey DR , Latz E and Fitzgerald KA (2009) AIM2 recognizes cytosolic dsDNA and forms a caspase‐1‐activating inflammasome with ASC. Nature 458, 514–518. 19158675 10.1038/nature07725 PMC2726264

[130] Kang JY , Choi H , Oh JM , Kim M and Lee DC (2024) PM2.5 induces pyroptosis via activation of the ROS/NF‐κB signaling pathway in bronchial epithelial cells. Medicina (Mex) 60, 1434. 10.3390/medicina60091434 PMC11434086 39336475

[131] Hou J , Lei Z , Cui L , Hou Y , Yang L , An R , Wang Q , Li S , Zhang H and Zhang L (2021) Polystyrene microplastics lead to pyroptosis and apoptosis of ovarian granulosa cells via NLRP3/Caspase‐1 signaling pathway in rats. Ecotoxicol Environ Saf 212, 112012. 33550074 10.1016/j.ecoenv.2021.112012

[132] Zhu YT , Yuan YZ , Feng QP , Hu MY , Li WJ , Wu X , Xiang SY and Yu SQ (2021) Food emulsifier polysorbate 80 promotes the intestinal absorption of mono‐2‐ethylhexyl phthalate by disturbing intestinal barrier. Toxicol Appl Pharmacol 414, 115411. 33476678 10.1016/j.taap.2021.115411

[133] Wu MMH , Zhang H , Yang Y , Wang Y , Luk PKH , Xia IF , Wong KH and Kwok KWH (2024) Food emulsifiers aggravate inflammation and oxidative stress induced by food contaminants in zebrafish. Food Chem Toxicol 191, 114850. 38986831 10.1016/j.fct.2024.114850

[134] Fu P , Zhang M , Bai L , Chen S , Chen W , Li Z , Yue J , Dong C and Li R (2025) Intestinal bacterial dysbiosis and liver fibrosis in mice through gut‐liver Axis and NLRP3 inflammatory pathway caused by fine particulate matter. J Appl Toxicol 45, 1030–1042. 39979029 10.1002/jat.4767

[135] Panyod S , Wu WK , Chang CT , Wada N , Ho HC , Lo YL , Tsai SP , Chen RA , Huang HS , Liu PY *et al*. (2024) Common dietary emulsifiers promote metabolic disorders and intestinal microbiota dysbiosis in mice. Commun Biol 7, 1–14. 38902371 10.1038/s42003-024-06224-3 PMC11190199

[136] Xu R , Cao J w , Lv H l , Geng Y and Guo M y (2024) Polyethylene microplastics induced gut microbiota dysbiosis leading to liver injury via the TLR2/NF‐κB/NLRP3 pathway in mice. Sci Total Environ 917, 170518. 38286276 10.1016/j.scitotenv.2024.170518

[137] Torices S , Daire L , Simon S , Mendoza L , Daniels D , Joseph JA , Fattakhov N , Naranjo O , Teglas T and Toborek M (2023) The NLRP3 inflammasome and gut dysbiosis as a putative link between HIV‐1 infection and ischemic stroke. Trends Neurosci 46, 682–693. 37330380 10.1016/j.tins.2023.05.009 PMC10554647

[138] Zmora N , Levy M , Pevsner‐Fishcer M and Elinav E (2017) Inflammasomes and intestinal inflammation. Mucosal Immunol 10, 865–883. 28401932 10.1038/mi.2017.19

[139] Pat Y , Yazici D , D'Avino P , Li M , Ardicli S , Ardicli O , Mitamura Y , Akdis M , Dhir R , Nadeau K *et al*. (2024) Recent advances in the epithelial barrier theory. Int Immunol 36, 211–222. 38227765 10.1093/intimm/dxae002 PMC10989673

[140] Kang R , Li R , Dai P , Li Z , Li Y and Li C (2019) Deoxynivalenol induced apoptosis and inflammation of IPEC‐J2 cells by promoting ROS production. Environ Pollut 251, 689–698. 31108302 10.1016/j.envpol.2019.05.026

[141] Serini S , Cassano R , Facchinetti E , Amendola G , Trombino S and Calviello G (2019) Anti‐irritant and anti‐inflammatory effects of DHA encapsulated in resveratrol‐based solid lipid nanoparticles in human keratinocytes. Nutrients 11, 1400. 31234344 10.3390/nu11061400 PMC6627705

[142] Dostert C , Pétrilli V , Van Bruggen R , Steele C , Mossman BT and Tschopp J (2008) Innate immune activation through Nalp3 inflammasome sensing of asbestos and silica. Science 320, 674–677. 18403674 10.1126/science.1156995 PMC2396588

[143] Martinou JC and Youle RJ (2011) Mitochondria in apoptosis: Bcl‐2 family members and mitochondrial dynamics. Dev Cell 21, 92–101. 21763611 10.1016/j.devcel.2011.06.017 PMC3156409

[144] Bertheloot D , Latz E and Franklin BS (2021) Necroptosis, pyroptosis and apoptosis: an intricate game of cell death. Cell Mol Immunol 18, 1106–1121. 33785842 10.1038/s41423-020-00630-3 PMC8008022

[145] Singh R , Letai A and Sarosiek K (2019) Regulation of apoptosis in health and disease: the balancing act of BCL‐2 family proteins. Nat Rev Mol Cell Biol 20, 175–193. 30655609 10.1038/s41580-018-0089-8 PMC7325303

[146] Chehab NH , Malikzay A , Stavridi ES and Halazonetis TD (1999) Phosphorylation of Ser‐20 mediates stabilization of human p53 in response to DNA damage. Proc Natl Acad Sci USA 96, 13777–13782. 10570149 10.1073/pnas.96.24.13777 PMC24141

[147] Ticli G , Cazzalini O , Stivala LA and Prosperi E (2022) Revisiting the function of p21CDKN1A in DNA repair: the influence of protein interactions and stability. Int J Mol Sci 23, 7058. 35806061 10.3390/ijms23137058 PMC9267019

[148] Aubrey BJ , Kelly GL , Janic A , Herold MJ and Strasser A (2018) How does p53 induce apoptosis and how does this relate to p53‐mediated tumour suppression? Cell Death Differ 25, 104–113. 29149101 10.1038/cdd.2017.169 PMC5729529

[149] Tabas I and Ron D (2011) Integrating the mechanisms of apoptosis induced by endoplasmic reticulum stress. Nat Cell Biol 13, 184–190. 21364565 10.1038/ncb0311-184 PMC3107571

[150] Liu T , Hou B , Wang Z and Yang Y (2022) Polystyrene microplastics induce mitochondrial damage in mouse GC‐2 cells. Ecotoxicol Environ Saf 237, 113520. 35489138 10.1016/j.ecoenv.2022.113520

[151] Lee SE , Yi Y , Moon S , Yoon H and Park YS (2022) Impact of micro‐ and nanoplastics on mitochondria. Metabolites 12, 897. 36295799 10.3390/metabo12100897 PMC9612075

[152] An Z , Liu G , Shen L , Qi Y , Hu Q , Song J , Li J , du J , Bai Y and Wu W (2024) Mitochondrial dysfunction induced by ambient fine particulate matter and potential mechanisms. Environ Res 262, 119930. 39237017 10.1016/j.envres.2024.119930

[153] Kalkavan H and Green DR (2018) MOMP, cell suicide as a BCL‐2 family business. Cell Death Differ 25, 46–55. 29053143 10.1038/cdd.2017.179 PMC5729535

[154] Srinivas US , Tan BWQ , Vellayappan BA and Jeyasekharan AD (2018) ROS and the DNA damage response in cancer. Redox Biol 25, 101084. 30612957 10.1016/j.redox.2018.101084 PMC6859528

[155] Moin I , Mittal D and Verma AK (2022) Understanding ROS‐induced DNA damage for therapeutics. In Handbook of Oxidative Stress in Cancer: Mechanistic Aspects ( Chakraborti S , Ray BK and Roychoudhury S , eds), pp. 897–918. Springer Nature, Singapore.

[156] Juan CA , Pérez de la Lastra JM , Plou FJ and Pérez‐Lebeña E (2021) The chemistry of reactive oxygen species (ROS) revisited: outlining their role in biological macromolecules (DNA, lipids and proteins) and induced pathologies. Int J Mol Sci 22, 4642. 33924958 10.3390/ijms22094642 PMC8125527

[157] Zhao H , Wu L , Yan G , Chen Y , Zhou M , Wu Y and Li Y (2021) Inflammation and tumor progression: signaling pathways and targeted intervention. Signal Transduct Target Ther 6, 1–46. 34248142 10.1038/s41392-021-00658-5 PMC8273155

[158] Multhoff G , Molls M and Radons J (2012) Chronic inflammation in cancer development. Front Immunol 2, 98. 22566887 10.3389/fimmu.2011.00098 PMC3342348

[159] Ivanov AI , Parkos CA and Nusrat A (2010) Cytoskeletal regulation of epithelial barrier function during inflammation. Am J Pathol 177, 512–524. 20581053 10.2353/ajpath.2010.100168 PMC2913378

[160] Citalán‐Madrid AF , Vargas‐Robles H , García‐Ponce A , Shibayama M , Betanzos A , Nava P , Salinas‐Lara C , Rottner K , Mennigen R and Schnoor M (2017) Cortactin deficiency causes increased RhoA/ROCK1‐dependent actomyosin contractility, intestinal epithelial barrier dysfunction, and disproportionately severe DSS‐induced colitis. Mucosal Immunol 10, 1237–1247. 28120846 10.1038/mi.2016.136

[161] van den Goor L , Iseler J , Koning KM and Miller AL (2024) Mechanosensitive recruitment of vinculin maintains junction integrity and barrier function at epithelial tricellular junctions. Curr Biol 34, 4677–4691.e5. 39341202 10.1016/j.cub.2024.08.060 PMC11496005

[162] Naydenov NG and Ivanov AI (2011) Spectrin‐adducin membrane skeleton: a missing link between epithelial junctions and the actin cytoskeletion? BioArchitecture 1, 186–191. 22069512 10.4161/bioa.1.4.17642 PMC3210521

[163] Yu H , Luo C , Linghu R , Yang J and Wu H (2024) Ezrin contributes to the damage of airway epithelial barrier related to diabetes mellitus. J Inflamm Res 17, 2609–2621. 38689797 10.2147/JIR.S449487 PMC11060175

[164] Shiomi R , Shigetomi K , Inai T , Sakai M and Ikenouchi J (2015) CaMKII regulates the strength of the epithelial barrier. Sci Rep 5, 13262. 26281891 10.1038/srep13262 PMC4539604

[165] Saatian B , Rezaee F , Desando S , Emo J , Chapman T , Knowlden S and Georas SN (2013) Interleukin‐4 and interleukin‐13 cause barrier dysfunction in human airway epithelial cells. Tissue Barriers 1, e24333. 24665390 10.4161/tisb.24333 PMC3875607

[166] Wu L , Oshima T , Li M , Tomita T , Fukui H , Watari J and Miwa H (2018) Filaggrin and tight junction proteins are crucial for IL‐13‐mediated esophageal barrier dysfunction. Am J Physiol Gastrointest Liver Physiol 315, G341–G350. 29746170 10.1152/ajpgi.00404.2017

[167] Macias‐Ceja DC , Mendoza‐Ballesteros MT , Ortega‐Albiach M , Barrachina MD and Ortiz‐Masià D (2023) Role of the epithelial barrier in intestinal fibrosis associated with inflammatory bowel disease: relevance of the epithelial‐to mesenchymal transition. Front Cell Dev Biol 11, 1258843. 37822869 10.3389/fcell.2023.1258843 PMC10562728

[168] Mottais A , Riberi L , Falco A , Soccal S , Gohy S and De Rose V (2023) Epithelial–mesenchymal transition mechanisms in chronic airway diseases: a common process to target? Int J Mol Sci 24, 12412. 37569787 10.3390/ijms241512412 PMC10418908

[169] Kitazawa K , Tanaka K , Kubota Y , Musashi M , Higashi K , Nagasawa T , Kobayashi M , Kamakura T , Igarashi R and Yamaguchi Y (2024) Expression of epithelial–mesenchymal transition markers in epidermal layer of atopic dermatitis. Biol Pharm Bull 47, 49–59. 38171779 10.1248/bpb.b23-00291

[170] Yan C , Grimm WA , Garner WL , Qin L , Travis T , Tan N and Han YP (2010) Epithelial to mesenchymal transition in human skin wound healing is induced by tumor necrosis factor‐α through bone Morphogenic Protein‐2. Am J Pathol 176, 2247–2258. 20304956 10.2353/ajpath.2010.090048 PMC2861090

[171] Sistigu A , Di Modugno F , Manic G and Nisticò P (2017) Deciphering the loop of epithelial‐mesenchymal transition, inflammatory cytokines and cancer immunoediting. Cytokine Growth Factor Rev 36, 67–77. 28595838 10.1016/j.cytogfr.2017.05.008

[172] Anderson ED , Alishahedani ME and Myles IA (2020) Epithelial‐mesenchymal transition in atopy: a mini‐review. Front Allergy 1, 628381. 34308414 10.3389/falgy.2020.628381 PMC8301597

[173] Heller F , Florian P , Bojarski C , Richter J , Christ M , Hillenbrand B , Mankertz J , Gitter AH , Bürgel N , Fromm M *et al*. (2005) Interleukin‐13 is the key effector Th2 cytokine in ulcerative colitis that affects epithelial tight junctions, apoptosis, and cell restitution. Gastroenterology 129, 550–564. doi: 10.1053/j.gastro.2005.05.002 16083712

[174] Wang F , Graham WV , Wang Y , Witkowski ED , Schwarz BT and Turner JR (2005) Interferon‐γ and tumor necrosis factor‐α synergize to induce intestinal epithelial barrier dysfunction by up‐regulating myosin light chain kinase expression. Am J Pathol 166, 409–419. 15681825 10.1016/s0002-9440(10)62264-x PMC1237049

[175] Wan H , Winton HL , Soeller C , Tovey ER , Gruenert DC , Thompson PJ , Stewart GA , Taylor GW , Garrod DR , Cannell MB *et al*. (1999) Der p 1 facilitates transepithelial allergen delivery by disruption of tight junctions. J Clin Invest 104, 123–133. 10393706 10.1172/JCI5844 PMC408401

[176] Gruber R , Börnchen C , Rose K , Daubmann A , Volksdorf T , Wladykowski E , Vidal‐y‐Sy S , Peters EM , Danso M , Bouwstra JA *et al*. (2015) Diverse regulation of claudin‐1 and claudin‐4 in atopic dermatitis. Am J Pathol 185, 2777–2789. 26319240 10.1016/j.ajpath.2015.06.021

[177] Krug SM , Amasheh S , Richter JF , Milatz S , Günzel D , Westphal JK , Huber O , Schulzke JD and Fromm M (2009) Tricellulin forms a barrier to macromolecules in tricellular tight junctions without affecting ion permeability. Mol Biol Cell 20, 3713–3724. 19535456 10.1091/mbc.E09-01-0080 PMC2777931

[178] Krug SM , Bojarski C , Fromm A , Lee IM , Dames P , Richter JF , Turner JR , Fromm M and Schulzke JD (2018) Tricellulin is regulated via interleukin‐13‐receptor α2, affects macromolecule uptake, and is decreased in ulcerative colitis. Mucosal Immunol 11, 345–356. 28612843 10.1038/mi.2017.52 PMC5730503

[179] Marchiando AM , Shen L , Graham WV , Edelblum KL , Duckworth CA , Guan Y , Montrose MH , Turner JR and Watson AJM (2011) The epithelial barrier is maintained by in vivo tight junction expansion during pathologic intestinal epithelial shedding. Gastroenterology 140, 1208–1218.e2. 21237166 10.1053/j.gastro.2011.01.004 PMC3066304

[180] Sherrill JD , Kc K , Wu D , Djukic Z , Caldwell JM , Stucke EM , Kemme KA , Costello MS , Mingler MK , Blanchard C *et al*. (2014) Desmoglein‐1 regulates esophageal epithelial barrier function and immune responses in eosinophilic esophagitis. Mucosal Immunol 7, 718–729. 24220297 10.1038/mi.2013.90 PMC3999291

[181] Costan VV , Popa C , Hâncu MF , Porumb‐Andrese E and Toader MP (2021) Comprehensive review on the pathophysiology, clinical variants and management of pemphigus (review). Exp Ther Med 22, 1335. 34630689 10.3892/etm.2021.10770 PMC8495539

[182] Akdis CA , Arkwright PD , Brüggen MC , Busse W , Gadina M , Guttman‐Yassky E , Kabashima K , Mitamura Y , Vian L , Wu J *et al*. (2020) Type 2 immunity in the skin and lungs. Allergy 75, 1582–1605. 32319104 10.1111/all.14318

[183] Iemoli E , Trabattoni D , Parisotto S , Borgonovo L , Toscano M , Rizzardini G , Clerici M , Ricci E , Fusi A , de Vecchi E *et al*. (2012) Probiotics reduce gut microbial translocation and improve adult atopic dermatitis. J Clin Gastroenterol 46, S33–S40. 22955355 10.1097/MCG.0b013e31826a8468

[184] Zheng D , Liwinski T and Elinav E (2020) Interaction between microbiota and immunity in health and disease. Cell Res 30, 492–506. 32433595 10.1038/s41422-020-0332-7 PMC7264227

[185] Bachert C , Gevaert P , Holtappels G , Johansson SGO and Cauwenberge PV (2001) Total and specific IgE in nasal polyps is related to local eosinophilic inflammation. J Allergy Clin Immunol 107, 607–614. 11295647 10.1067/mai.2001.112374

[186] Sintobin I , Siroux V , Holtappels G , Pison C , Nadif R , Bousquet J and Bachert C (2019) Sensitisation to staphylococcal enterotoxins and asthma severity: a longitudinal study in the EGEA cohort. Eur Respir J 54, 1900198. 31285304 10.1183/13993003.00198-2019

[187] Sørensen MV , Klingenberg C , Wickman M , Sollid JUE , Furberg AS , Bachert C and Bousquet J (2017) *Staphylococcus aureus* enterotoxin sensitization is associated with allergic poly‐sensitization and allergic multimorbidity in adolescents. Allergy 72, 1548–1555. 28378344 10.1111/all.13175

[188] Friedman SJ , Schroeter AL and Homburger HA (1985) IgE antibodies to *Staphylococcus aureus* . Arch Dermatol 121, 869. 4015132

[189] Kim YC , Won HK , Lee JW , Sohn KH , Kim MH , Kim TB , Chang YS , Lee BJ , Cho SH , Bachert C *et al*. (2019) Staphylococcus aureus nasal colonization and asthma in adults: systematic review and meta‐analysis. J Allergy Clin Immunol Pract 7, 606–615.e9. 30193937 10.1016/j.jaip.2018.08.020

[190] Meng J , Xiao H , Xu F , She X , Liu C and Canonica GW (2025) Systemic barrier dysfunction in type 2 inflammation diseases: perspective in the skin, airways, and gastrointestinal tract. Immunol Res 73, 60. 40069459 10.1007/s12026-025-09606-9 PMC11897119

[191] Orsmond A , Bereza‐Malcolm L , Lynch T , March L and Xue M (2021) Skin barrier dysregulation in psoriasis. Int J Mol Sci 22, 10841. 34639182 10.3390/ijms221910841 PMC8509518

[192] Chauvin C , Retnakumar SV and Bayry J (2023) Gasdermin D as a cellular switch to orientate immune responses via IL‐33 or IL‐1β. Cell Mol Immunol 20, 8–10. 36380096 10.1038/s41423-022-00950-6 PMC9664042

[193] Ohara D , Takeuchi Y and Hirota K (2024) Type 17 immunity: novel insights into intestinal homeostasis and autoimmune pathogenesis driven by gut‐primed T cells. Cell Mol Immunol 21, 1183–1200. 39379604 10.1038/s41423-024-01218-x PMC11528014

[194] Utech M , Ivanov AI , Samarin SN , Bruewer M , Turner JR , Mrsny RJ , Parkos CA and Nusrat A (2005) Mechanism of IFN‐γ‐induced endocytosis of tight junction proteins: myosin II‐dependent vacuolarization of the apical plasma membrane. Mol Biol Cell 16, 5040–5052. 16055505 10.1091/mbc.E05-03-0193 PMC1237102

[195] Lal M , Burk CM , Gautam R , Mrozek Z , Canziani KE , Trachsel T , Beers J , Carroll MC , Morgan DM , Muir AB *et al*. (2025) Interferon‐γ signaling in eosinophilic esophagitis affects epithelial barrier function and programmed cell death. Cell Mol Gastroenterol Hepatol 19, 101466. 39884574 10.1016/j.jcmgh.2025.101466 PMC11964763

[196] Davis BP , Stucke EM , Khorki ME , Litosh VA , Rymer JK , Rochman M , Travers J , Kottyan LC and Rothenberg ME (2016) Eosinophilic esophagitis–linked calpain 14 is an IL‐13–induced protease that mediates esophageal epithelial barrier impairment. JCI Insight 1, e86355. 27158675 10.1172/jci.insight.86355 PMC4855700

[197] Furue M and Furue M (2021) Interleukin‐22 and keratinocytes; pathogenic implications in skin inflammation. Explor Immunol 1, 37–47.

[198] Yazici D , Pat Y , Mitamura Y , Akdis CA and Ogulur I (2023) Detergent‐induced eosinophilic inflammation in the esophagus: a key evidence for the epithelial barrier theory. Allergy 78, 1422–1424. 36645729 10.1111/all.15646

[199] Stefanovic N , Flohr C and Irvine AD (2020) The exposome in atopic dermatitis. Allergy 75, 63–74. 31194890 10.1111/all.13946 PMC7003958

[200] Hui‐Beckman J , Kim BE and Leung DY (2021) Origin of allergy from in utero exposures to the postnatal environment. Allergy, Asthma Immunol Res 14, 8–20. 10.4168/aair.2022.14.1.8 PMC8724834 34983104

[201] Boguniewicz M and Leung DYM (2011) Atopic dermatitis: a disease of altered skin barrier and immune dysregulation. Immunol Rev 242, 233–246. 21682749 10.1111/j.1600-065X.2011.01027.x PMC3122139

[202] Ranjbar M , Whetstone CE , Omer H , Power L , Cusack RP and Gauvreau GM (2022) The genetic factors of the airway epithelium associated with the pathology of asthma. Genes 13, 1870. 36292755 10.3390/genes13101870 PMC9601469

[203] Vercelli D (2003) Genetic polymorphism in allergy and asthma. Curr Opin Immunol 15, 609–613. 14630192 10.1016/j.coi.2003.09.005

[204] Shoda T , Kaufman KM , Wen T , Caldwell JM , Osswald GA , Purnima P , Zimmermann N , Collins MH , Rehn K , Foote H *et al*. (2021) Desmoplakin and periplakin genetically and functionally contribute to eosinophilic esophagitis. Nat Commun 12, 6795. 34815391 10.1038/s41467-021-26939-9 PMC8611043

[205] Klee KMC , Janecke AR , Civan HA , Rosipal Š , Heinz‐Erian P , Huber LA , Müller T and Vogel GF (2020) AP1S1 missense mutations cause a congenital enteropathy via an epithelial barrier defect. Hum Genet 139, 1247–1259. 32306098 10.1007/s00439-020-02168-w PMC7497319

[206] Hadj‐Rabia S , Brideau G , Al‐Sarraj Y , Maroun RC , Figueres ML , Leclerc‐Mercier S , Olinger E , Baron S , Chaussain C , Nochy D *et al*. (2018) Multiplex epithelium dysfunction due to CLDN10 mutation: the HELIX syndrome. Genet Med 20, 190–201. 28771254 10.1038/gim.2017.71

[207] Sivagnanam M , Mueller JL , Lee H , Chen Z , Nelson SF , Turner D , Zlotkin SH , Pencharz PB , Ngan B–Y , Libiger O *et al*. (2008) Identification of EpCAM as the gene for congenital tufting enteropathy. Gastroenterology 135, 429–437. 18572020 10.1053/j.gastro.2008.05.036 PMC2574708

[208] Vercelli D and Bleecker ER (2019) Strength in numbers: the quest for asthma genes. J Allergy Clin Immunol 144, 413–415. 31229462 10.1016/j.jaci.2019.06.007

[209] Gao W , Gong J , Mu M , Zhu Y , Wang W , Chen W , Han G , Hu H and Bao P (2021) The pathogenesis of eosinophilic asthma: a positive feedback mechanism that promotes Th2 immune response via Filaggrin deficiency. Front Immunol 12, 672312. 34484176 10.3389/fimmu.2021.672312 PMC8414997

[210] Wawrzyniak P , Krawczyk K , Acharya S , Tan G , Wawrzyniak M , Karouzakis E , Dreher A , Jakiela B , Altunbulakli C , Sanak M *et al*. (2021) Inhibition of CpG methylation improves the barrier integrity of bronchial epithelial cells in asthma. Allergy 76, 1864–1868. 33210726 10.1111/all.14667

[211] Daccache JA and Naik S (2024) Inflammatory memory in chronic skin disease. JID Innov 4, 100277. 38708420 10.1016/j.xjidi.2024.100277 PMC11068922

[212] Surace AEA and Hedrich CM (2019) The role of epigenetics in autoimmune/inflammatory disease. Front Immunol 10, 1525. 31333659 10.3389/fimmu.2019.01525 PMC6620790

[213] Geoghegan JA , Irvine AD and Foster TJ (2018) *Staphylococcus aureus* and atopic dermatitis: a complex and evolving relationship. Trends Microbiol 26, 484–497. 29233606 10.1016/j.tim.2017.11.008

[214] Moffatt MF and Cookson W (2017) The lung microbiome in health and disease. Clin Med 17, 525–529. 10.7861/clinmedicine.17-6-525 PMC6297685 29196353

[215] Nakatsuji T , Chen TH , Narala S , Chun KA , Two AM , Yun T , Shafiq F , Kotol PF , Bouslimani A , Melnik AV *et al*. (2017) Antimicrobials from human skin commensal bacteria protect against *Staphylococcus aureus* and are deficient in atopic dermatitis. Sci Transl Med 9, eaah4680. 28228596 10.1126/scitranslmed.aah4680 PMC5600545

[216] Ha C , Martin A , Sepich‐Poore GD , Shi B , Wang Y , Gouin K , Humphrey G , Sanders K , Ratnayake Y , Chan KSL *et al*. (2020) Translocation of viable gut microbiota to mesenteric adipose drives formation of creeping fat in humans. Cell 183, 666–683.e17. 32991841 10.1016/j.cell.2020.09.009 PMC7521382

[217] Dinh DM , Volpe GE , Duffalo C , Bhalchandra S , Tai AK , Kane AV , Wanke CA and Ward HD (2015) Intestinal microbiota, microbial translocation, and systemic inflammation in chronic HIV infection. J Infect Dis 211, 19–27. 25057045 10.1093/infdis/jiu409 PMC4326316

[218] Sandler NG and Douek DC (2012) Microbial translocation in HIV infection: causes, consequences and treatment opportunities. Nat Rev Microbiol 10, 655–666. 22886237 10.1038/nrmicro2848

[219] Brenchley JM , Price DA , Schacker TW , Asher TE , Silvestri G , Rao S , Kazzaz Z , Bornstein E , Lambotte O , Altmann D *et al*. (2006) Microbial translocation is a cause of systemic immune activation in chronic HIV infection. Nat Med 12, 1365–1371. 17115046 10.1038/nm1511

[220] Kumar NP , Venkataraman A , Hanna LE , Putlibai S , Karthick M , Rajamanikam A , Sadasivam K , Sundaram B and Babu S (2021) Systemic inflammation and microbial translocation are characteristic features of SARS‐CoV‐2‐related multisystem inflammatory syndrome in children. Open Forum Infect Dis 8, ofab279. 34322566 10.1093/ofid/ofab279 PMC8312521

[221] Sorini C , Cosorich I , Conte ML , Lo Conte M , De Giorgi L , Facciotti F , Lucianò R , Rocchi M , Ferrarese R , Sanvito F *et al*. (2019) Loss of gut barrier integrity triggers activation of islet‐reactive T cells and autoimmune diabetes. Proc Natl Acad Sci USA 116, 15140–15149. 31182588 10.1073/pnas.1814558116 PMC6660755

[222] Gupta B , Rai R , Oertel M and Raeman R (2022) Intestinal barrier dysfunction in fatty liver disease: roles of microbiota, mucosal immune system, and bile acids. Semin Liver Dis 42, 122–137. 35738255 10.1055/s-0042-1748037 PMC9307091

[223] Yang Y , Nguyen M , Khetrapal V , Sonnert ND , Martin AL , Chen H , Kriegel MA and Palm NW (2022) Within‐host evolution of a gut pathobiont facilitates liver translocation. Nature 607, 563–570. 35831502 10.1038/s41586-022-04949-x PMC9308686

[224] Tilg H , Zmora N , Adolph TE and Elinav E (2020) The intestinal microbiota fuelling metabolic inflammation. Nat Rev Immunol 20, 40–54. 31388093 10.1038/s41577-019-0198-4

[225] Belkaid Y and Hand TW (2014) Role of the microbiota in immunity and inflammation. Cell 157, 121–141. 24679531 10.1016/j.cell.2014.03.011 PMC4056765

[226] Rath E and Haller D (2022) Intestinal epithelial cell metabolism at the interface of microbial dysbiosis and tissue injury. Mucosal Immunol 15, 595–604. 35534699 10.1038/s41385-022-00514-x PMC9259489

[227] Mues N and Chu HW (2020) Out‐smarting the host: bacteria maneuvering the immune response to favor their survival. Front Immunol 11, 819. 32477341 10.3389/fimmu.2020.00819 PMC7235365

[228] Dey P (2024) Good girl goes bad: understanding how gut commensals cause disease. Microb Pathog 190, 106617. 38492827 10.1016/j.micpath.2024.106617

[229] Howden BP , Giulieri SG , Wong Fok Lung T , Baines SL , Sharkey LK , Lee JYH , Hachani A , Monk IR and Stinear TP (2023) *Staphylococcus aureus* host interactions and adaptation. Nat Rev Microbiol 21, 380–395. 36707725 10.1038/s41579-023-00852-y PMC9882747

[230] Kretschmer D , Breitmeyer R , Gekeler C , Lebtig M , Schlatterer K , Nega M , Stahl M , Stapels D , Rooijakkers S and Peschel A (2021) *Staphylococcus aureus* depends on Eap proteins for preventing degradation of its phenol‐soluble modulin toxins by neutrophil serine proteases. Front Immunol 12, 701093. 34552584 10.3389/fimmu.2021.701093 PMC8451722

[231] Parrish A , Boudaud M , Grant ET , Willieme S , Neumann M , Wolter M , Craig SZ , de Sciscio A , Cosma A , Hunewald O *et al*. (2023) *Akkermansia muciniphila* exacerbates food allergy in fibre‐deprived mice. Nat Microbiol 8, 1863–1879. 37696941 10.1038/s41564-023-01464-1 PMC10522492

[232] Fung C , Fraser LM , Barrón GM , Gologorsky MB , Atkinson SN , Gerrick ER , Hayward M , Ziegelbauer J , Li JA , Nico KF *et al*. (2023) Tuft cells mediate commensal remodeling of the small intestinal antimicrobial landscape. Proc Natl Acad Sci USA 120, e2216908120. 37253002 10.1073/pnas.2216908120 PMC10266004

[233] Ruchti F , Zwicky P , Becher B , Dubrac S and LeibundGut‐Landmann S (2024) Epidermal barrier impairment predisposes for excessive growth of the allergy‐associated yeast *Malassezia* on murine skin. Allergy 79, 1531–1547. 38385963 10.1111/all.16062

[234] Kanj AN , Kottom TJ , Schaefbauer KJ , Choudhury M , Limper AH and Skalski JH (2023) Dysbiosis of the intestinal fungal microbiota increases lung resident group 2 innate lymphoid cells and is associated with enhanced asthma severity in mice and humans. Respir Res 24, 144. 37259076 10.1186/s12931-023-02422-5 PMC10230676

[235] Dokoshi T , Chen Y , Cavagnero KJ , Rahman G , Hakim D , Brinton S , Schwarz H , Brown EA , O'Neill A , Nakamura Y *et al*. (2024) Dermal injury drives a skin to gut axis that disrupts the intestinal microbiome and intestinal immune homeostasis in mice. Nat Commun 15, 3009. 38589392 10.1038/s41467-024-47072-3 PMC11001995

[236] Agaronyan K , Sharma L , Vaidyanathan B , Glenn K , Yu S , Annicelli C , Wiggen TD , Penningroth MR , Hunter RC , dela Cruz CS *et al*. (2022) Tissue remodeling by an opportunistic pathogen triggers allergic inflammation. Immunity 55, 895–911.e10. 35483356 10.1016/j.immuni.2022.04.001 PMC9123649

[237] Wallen‐Russell C , Pearlman N , Wallen‐Russell S , Cretoiu D , Thompson DC and Voinea SC (2023) A catastrophic biodiversity loss in the environment is being replicated on the skin microbiome: is this a major contributor to the chronic disease epidemic? Microorganisms 11, 2784. 38004795 10.3390/microorganisms11112784 PMC10672968

[238] Kriss M , Hazleton KZ , Nusbacher NM , Martin CG and Lozupone CA (2018) Low diversity gut microbiota dysbiosis: drivers, functional implications and recovery. Curr Opin Microbiol 44, 34–40. 30036705 10.1016/j.mib.2018.07.003 PMC6435260

[239] Ryguła I , Pikiewicz W , Grabarek BO , Wójcik M and Kaminiów K (2024) The role of the gut microbiome and microbial dysbiosis in common skin diseases. Int J Mol Sci 25, 1984. 38396663 10.3390/ijms25041984 PMC10889245

[240] Hashim A , Alsam A , Payne MA , Aduse‐Opoku J , Curtis MA and Joseph S (2021) Loss of neutrophil homing to the periodontal tissues modulates the composition and disease potential of the oral microbiota. Infect Immun 89, e0030921. 34491788 10.1128/IAI.00309-21 PMC8594596

[241] Qi‐Xiang M , Yang F , Huang Z‐H , Nuo‐Ming Y , Rui‐Long W , Bin‐Qiang X , Jun‐Jie F , Chun‐Lan H and Yue Z (2022) Intestinal TLR4 deletion exacerbates acute pancreatitis through gut microbiota dysbiosis and Paneth cells deficiency. Gut Microbes 14, 2112882. 35982604 10.1080/19490976.2022.2112882 PMC9397436

[242] Long SR , Shang WX , Zhang HR , Jiang M , Wang JJ , Liu RD , Wang ZQ , Cui J and Sun H (2024) *Trichinella‐*derived protein ameliorates colitis by altering the gut microbiome and improving intestinal barrier function. Int Immunopharmacol 127, 111320. 38064817 10.1016/j.intimp.2023.111320

[243] Masuda MY , Pyon GC , Luo H , LeSuer WE , Putikova A , Dao A , Ortiz DR , Schulze AR , Fritz N , Kobayashi T *et al*. (2024) Epithelial overexpression of IL‐33 induces eosinophilic esophagitis dependent on IL‐13. J Allergy Clin Immunol 153, 1355–1368. 38310974 10.1016/j.jaci.2024.01.017 PMC11070306

[244] Zhou X , Sampath V and Nadeau KC (2024) Effect of air pollution on asthma. Ann Allergy Asthma Immunol 132, 426–432. 38253122 10.1016/j.anai.2024.01.017 PMC10990824

[245] Bachert C , Hicks A , Gane S , Peters AT , Gevaert P , Nash S , Horowitz JE , Sacks H and Jacob‐Nara JA (2024) The interleukin‐4/interleukin‐13 pathway in type 2 inflammation in chronic rhinosinusitis with nasal polyps. Front Immunol 15, 1356298. 38690264 10.3389/fimmu.2024.1356298 PMC11059040

[246] Werder RB , Ullah MA , Rahman MM , Simpson J , Lynch JP , Collinson N , Rittchen S , Rashid RB , Sikder MAA , Handoko HY *et al*. (2022) Targeting the P2Y13 receptor suppresses IL‐33 and HMGB1 release and ameliorates experimental asthma. Am J Respir Crit Care Med 205, 300–312. 34860143 10.1164/rccm.202009-3686OC PMC12042653

[247] Nazari H , Shrestha J , Naei VY , Bazaz SR , Sabbagh M , Thiery JP and Warkiani ME (2023) Advances in TEER measurements of biological barriers in microphysiological systems. Biosens Bioelectron 234, 115355. 37159988 10.1016/j.bios.2023.115355

[248] Gan J , Nazarian S , Teare J , Darzi A , Ashrafian H and Thompson AJ (2022) A case for improved assessment of gut permeability: a meta‐analysis quantifying the lactulose:mannitol ratio in coeliac and Crohn's disease. BMC Gastroenterol 22, 16. 35012471 10.1186/s12876-021-02082-z PMC8751358

[249] Rath T , Dieterich W , Kätscher‐Murad C , Neurath MF and Zopf Y (2021) Cross‐sectional imaging of intestinal barrier dysfunction by confocal laser endomicroscopy can identify patients with food allergy in vivo with high sensitivity. Sci Rep 11, 12777. 34140591 10.1038/s41598-021-92262-4 PMC8211682

[250] Rinaldi AO , Morita H , Wawrzyniak P , Dreher A , Grant S , Svedenhag P and Akdis CA (2019) Direct assessment of skin epithelial barrier by electrical impedance spectroscopy. Allergy 74, 1934–1944. 30989659 10.1111/all.13824

[251] Rinaldi AO , Korsfeldt A , Ward S , Burla D , Dreher A , Gautschi M , Stolpe B , Tan G , Bersuch E , Melin D *et al*. (2021) Electrical impedance spectroscopy for the characterization of skin barrier in atopic dermatitis. Allergy 76, 3066–3079. 33830511 10.1111/all.14842

[252] Huygen L , Thys PM , Wollenberg A , Gutermuth J and Krohn IK (2024) Skin barrier function assessment: electrical impedance spectroscopy is less influenced by daily routine activities than Transepidermal water loss. Ann Dermatol 36, 99–111. 38576248 10.5021/ad.23.052 PMC10995614

[253] Marshall‐Webb M , Myers JC , Watson DI , Bright T , Omari TI and Thompson SK (2024) Mucosal impedance as a diagnostic tool for gastroesophageal reflux disease: an update for clinicians. Dis Esophagus 37, doae037. 38670809 10.1093/dote/doae037 PMC11360985

[254] Zhang Q , Lang Y , Tang X , Cheng W , Cheng Z , Rizwan M , Xie L , Liu Y , Xu H and Liu Y (2024) Polystyrene microplastic‐induced endoplasmic reticulum stress contributes to growth plate endochondral ossification disorder in young rat. Environ Toxicol 39, 3314–3329. 38440912 10.1002/tox.24182

